# Carbon sources and XlnR-dependent transcriptional landscape of CAZymes in the industrial fungus *Talaromyces versatilis*: when exception seems to be the rule

**DOI:** 10.1186/s12934-019-1062-8

**Published:** 2019-01-28

**Authors:** Agustina Llanos, Sébastien Déjean, Virginie Neugnot-Roux, Jean M. François, Jean-Luc Parrou

**Affiliations:** 1 LISBP, Université de Toulouse, INSA, INRA, CNRS, Toulouse, France; 2 Adisseo France S.A.S, 135 Avenue de Rangueil, 31077 Toulouse, France; 3 Institut de Mathématiques de Toulouse, UMR5219-Université de Toulouse; CNRS-UPS, 31062 Toulouse Cedex 9, France

**Keywords:** Filamentous fungi, Secretome, Glycosyl hydrolases, Cellulose, Hemicellulose, Biomass deconstruction, Transcription factors, XlnR/XYR1, Glucose repression, High-throughput qPCR

## Abstract

**Background:**

Research on filamentous fungi emphasized the remarkable redundancy in genes encoding hydrolytic enzymes, the similarities but also the large differences in their expression, especially through the role of the XlnR/XYR1 transcriptional activator. The purpose of this study was to evaluate the specificities of the industrial fungus *Talaromyces versatilis*, getting clues into the role of XlnR and the importance of glucose repression at the transcriptional level, to provide further levers for cocktail production.

**Results:**

By studying a set of 62 redundant genes representative of several categories of enzymes, our results underlined the huge plasticity of transcriptional responses when changing nutritional status. As a general trend, the more heterogeneous the substrate, the more efficient to trigger activation. Genetic modifications of *xlnR* led to significant reorganisation of transcriptional patterns. Just a minimal set of genes actually fitted in a simplistic model of regulation by a transcriptional activator, and this under specific substrates. On the contrary, the diversity of *xlnR*^+^ versus *ΔxlnR* responses illustrated the existence of complex and unpredicted patterns of co-regulated genes that were highly dependent on the culture condition, even between genes that encode members of a functional category of enzymes. They notably revealed a dual, substrate-dependant repressor-activator role of XlnR, with counter-intuitive transcripts regulations that targeted specific genes. About glucose, it appeared as a formal repressive sugar as we observed a massive repression of most genes upon glucose addition to the mycelium grown on wheat straw. However, we also noticed a positive role of this sugar on the basal expression of a few genes, (notably those encoding cellulases), showing again the strong dependence of these regulatory mechanisms upon promoter and nutritional contexts.

**Conclusions:**

The diversity of transcriptional patterns appeared to be the rule, while common and stable behaviour, both within gene families and with fungal literature, the exception. The setup of a new biotechnological process to reach optimized, if not customized expression patterns of enzymes, hence appeared tricky just relying on published data that can lead, in the best scenario, to approximate trends. We instead encourage preliminary experimental assays, carried out in the context of interest to reassess gene responses, as a mandatory step before thinking in (genetic) strategies for the improvement of enzyme production in fungi.
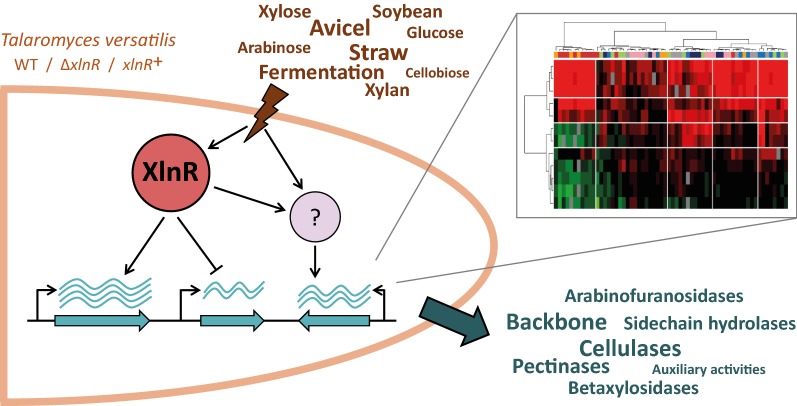

**Electronic supplementary material:**

The online version of this article (10.1186/s12934-019-1062-8) contains supplementary material, which is available to authorized users.

## Background

Biomass degradation is an essential yet complex process in nature, particularly for degrading the plant cell wall [1]. This structure is a highly heterogeneous material mainly composed of cellulose, hemicellulose, lignin and pectin, whose degradation is achieved through multiple means, including the use of enzymes with different and complementary activities [2–4]. The secretion of such a wide variety of enzymes is the hallmark of filamentous fungi [1, 5–8]. Part of these enzymes are designed ‘carbohydrate-active enzymes’ (CAZYmes) and are compiled into the Carbohydrate Active Enzymes database (http://www.cazy.org/; [9]). They are assigned into five enzyme classes, including Glycoside Hydrolases (GH) and Auxiliary Activities (AA). This classification relies on amino acids sequence similarity, secondary and tertiary fold conservation, and stereochemical architecture of catalytic mechanisms. This classification, however, does not preclude functional redundancy between families, as for instance enzymes bearing xylanase activity that belong to GH10, GH11, GH5, GH7, GH8 and GH43 families [10].

The ever-growing number of sequenced and annotated fungal genomes [11–24] revealed that fungi exhibit tremendous diversity in the number and variety of CAZymes [25–28]. Attempts to correlate growth profiles and gene content in fungal genomes, as for the pectinolytic system [4], indicated that the number of genes could correlate with the ability to degrade specific substrates. Mechanistically however, this correlation appeared drastically more complex when trying to decipher relations between pectin structural elements and the specific subsets of genes used for that deconstruction. From phylogeny viewpoint, the expansion or contraction of gene families is apparently largely lineage specific, and not shared among all fungi of a given lifestyle [29]. In fungi with highly enriched number of CAZymes, it was shown that the increase of GHs is not random across all GH families, but affects specific families to different degrees [18]. Unexpected traits could also be observed, as for plant pathogenic fungi that have in general the highest number of CAZymes while saprophytic fungi contain fewer, even if their high activity in degrading plant biomass would not predict such gene repertoire [28, 30, 31]. Also, the efficient cellulose-degrading fungi as the brown-rot fungi *Postia placenta* faced a significant loss of the gene repertoire typical of cellulolytic microbes, yet developed alternative oxidative mechanisms for biomass deconstruction [32]. Finally, as only a few genes from expanded multigene families were actively expressed in a given culture condition, and as it was different in different strains, it has been suggested that family expansion could increase adaptive opportunities rather than activity under a specific culture condition [33]. This extreme redundancy in gene family members and the diversity of their expression patterns, hence provide foundations for the extraordinary metabolic flexibility reported for filamentous fungi [34].

The filamentous fungus *Talaromyces versatilis*, (formerly *Penicillium funiculosum*), is exploited at the industrial scale to produce an enzymatic cocktail termed Rovabio^®^, which is used as feed additive to enhance the digestibility of cereals-based diets [35, 36] as it is particularly enriched in lignocellulolytic enzymes (i.e. xylanases, arabinofuranosidase, β-xylosidase, etc.) as well as proteases [37]. Independent studies on a *P.* *funiculosum* strain, also described the richness and hydrolytic potential of the secretome of this fungus when it is grown under different polymeric inducers [38, 39]. However, enzymes recovered in the Rovabio^®^ cocktail produced by *T. versatilis* represent only a fraction of the full set of CAZymes that could be in theory encoded by the genome of this fungus [37]. In total, 170 genes were manually annotated as GH or related enzymes, spread into 32 different GH families and relevant for the degradation of the plant polymers (unpublished, proprietary data). This is consistent with other fungal genome versus secretome comparisons, which showed that these microorganisms specifically express and secrete only a subset of CAZYmes, generally tailored to adapt to environmental resources [28, 33, 40–43]. This set of secreted proteins is most often species specific, as despite similar genomic potential of certain fungi, their approaches to degrade plant biomass indeed differ markedly in the overall patterns of regulation, activities as well as the specific sets of proteins they employ [32, 41, 44]. A recent, comparative transcriptomic analysis of *A. niger* and *T. reesei* grown on sugarcane bagasse confirmed that these fungi exhibited differences in the precise set of expressed genes and their expression profile, suggesting that they employ different strategies for biomass breakdown, even if a set of CAZymes such as cellulases, hemicellulases and oxidative enzymes was expressed by both fungal strains [45]. It was nevertheless shown that predicted secretomes of fungi with similar lifestyles shared certain characteristics, and not always correlated to gene repertoire and phylogenetic groups [33]. Expression of the secretome was for example largely influenced by the type of substrate or cultivation process [41, 46–48], growth development and pathogenic phase [30]. Also, key genomic mutations could significantly improve enzyme production [49]. All these data point to strict fine tuning of these sets of genes through sophisticated regulatory systems, to achieve optimal growth or adaptation to a given environmental condition [40], as for example in the model of sequential expression of the genes for an efficient degradation of the lignocellulose [50].

A handful of transcription factors (TF), characterized as activators or repressors of the expression of genes encoding CAZymes, have been isolated in different filamentous fungi (reviewed in [5, 34, 51–53]). Notably, CreA/CRE1 has been documented as the carbon catabolite transcriptional repressor of cellulolytic and xylanolytic genes in response to glucose. However, in filamentous fungi as *A. nidulans*, *N. crassa* and *T. reesei*, if CreA/Cre1/CRE1-dependent derepression occurs in the presence of complex carbohydrates such as lignocellulose, repression yet persists in response to high concentrations of alternative, simple carbon sources such as cellobiose and xylose, which results in CreA/Cre1 nuclear localisation in *A.* *nidulans* [54]. The transcription factor XlnR/XYR1 was originally reported as a xylanolytic regulator [55]. However, it was emphasized over time that large differences can be found in the sets of genes that are controlled by XlnR in different fungi species [56, 57]. In *M.* *oryzae* for example, it regulates the expression of genes involved in the pentose catabolic pathway, but not genes encoding hemi-cellulolytic enzymes [58].

The aim of this study was to delineate the role of the transcription factor XlnR in *T.* *versatilis* by the use of two mutant strains, *ΔxlnR* and *xlnR *^+^ (deletion and overexpression of the *xlnR* gene, respectively). We explored by high-throughput quantitative PCR the transcriptional landscape of 62 representative genes encoding CAZymes, in the wild type and the two *xlnR* mutant strains. *T. versatilis* was cultivated under a broad range of nutritional conditions, from simple sugars to more heterogeneous polymeric substrates, to evaluate further substrate-dependent transcriptional regulation of these genes, and especially the role of glucose.

## Results

### Balanced choice of GOIs to consider redundancy in the genes coding for similar hydrolytic activities in *T. versatilis*

**Fig. 1:**
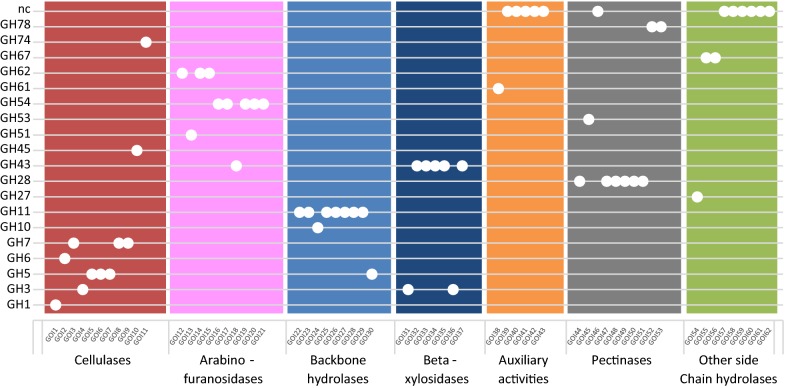
Affiliation of the 62 GOIs in the different functional categories and CAZY GH families. Functional categories (horizontal axis) and CAZY families (GH, vertical axis)

To better understand the transcriptional network that governs gene expression in the filamentous fungus *Talaromyces versatilis*, (formerly known as *Penicillium funiculosum*), we selected a set of 62 representative genes of interest (GOIs) encoding hydrolytic and auxiliary enzymes in plant biomass degradation (Table 1 and more detailed information in the Additional file 1). A preliminary list of GOIs was selected based on expressed candidates in a RNA-seq study carried out during cultures on glucose and wheat straw [59], and from a proteomic analysis of the Rovabio^®^ cocktail [37]. This list of expressed genes was extended to be more representative of several categories of enzymes. The category ‘cellulases’ gathered together 3 types of enzymes involved in the degradation of cellulose, namely endoglucanases, cellobiohydrolases and β-glucosidases. The categories ‘backbone hydrolases’, (mainly xylanases), ‘β-xylosidases’, ‘arabinofuranosidases’ and ‘other side chain hydrolases’, (mainly esterases), contained enzymes for hemicellulose degradation. Pectin is another important component of the plant cell wall and its degradation involves, among others, polygalacturonases and pectin lyases, which were included in the study under the category ‘pectinases’. Finally, we considered proteins that have no characterized enzymatic activity but are potentially involved in the degradation process, such as swollenins and hydrophobic binding proteins grouped under the category ‘auxiliary activities’. Figure 1 shows the affiliation of each gene to CAZY GH families. The ‘cellulases’ category notably illustrates the functional redundancy that can be found between GH families, with the six selected endoglucanases distributed in GH5, GH7, GH45 and GH74 families, the GH7 family also containing two of the three cellobiohydrolases.

**Table 1:** GOIs and their affiliation in the different functional categories

GOI #	CAZy family	Annotation/activity	Category
GOI1	GH1	β-Glucosidase	Cellulase
GOI2	GH6	CbhII	Cellulase
GOI3	GH7	CbhI	Cellulase
GOI4	GH3	β-glucosidase	Cellulase
GOI5	GH5	β-1,4-Endoglucanase	Cellulase
GOI6	GH5	β-1,4-Endoglucanase	Cellulase
GOI7	GH5	β-1,4-Endoglucanase	Cellulase
GOI8	GH7	Cellobiohydrolase (reducing end)	Cellulase
GOI9	GH7	β-1,4-Endoglucanase	Cellulase
GOI10	GH45	β-1,4-Endoglucanase	Cellulase
GOI11	GH74	Endoglucanase	Cellulase
GOI12	GH62	abf62a	Arabinofuranosidase
GOI13	GH51	abfA1	Arabinofuranosidase
GOI14	GH62	Abf62b	Arabinofuranosidase
GOI15	GH62	Abf62c	Arabinofuranosidase
GOI16	GH54	Abf-B2	Arabinofuranosidase
GOI17	GH54	Abf-B1	Arabinofuranosidase
GOI18	GH43	β-1,4-Xylosidase/α-arabinofuranosidase	Arabinofuranosidase
GOI19	GH54	α-l-Arabinofuranosidase	Arabinofuranosidase
GOI20	GH54	α-l-Arabinofuranosidase	Arabinofuranosidase
GOI21	GH54	α-l-Arabinofuranosidase	Arabinofuranosidase
GOI22	GH11	XynB	Backbone hydrolase
GOI23	GH11	XynC	Backbone hydrolase
GOI24	GH10	XynD	Backbone hydrolase
GOI25	GH11	XynE	Backbone hydrolase
GOI26	GH11	XynF	Backbone hydrolase
GOI27	GH11	XynG	Backbone hydrolase
GOI28	GH11	XynH	Backbone hydrolase
GOI29	GH11	XynI	Backbone hydrolase
GOI30	GH5	β-1,4-Endomannanase	Backbone hydrolase
GOI31	GH3	β-Xylosidase	Beta-xylosidase
GOI32	GH43	β-Xylosidase	Beta-xylosidase
GOI33	GH43	β-Xylosidase	Beta-xylosidase
GOI34	GH43	GH43	Beta-xylosidase
GOI35	GH43	GH43	Beta-xylosidase
GOI36	GH3	β-Xylosidase	Beta-xylosidase
GOI37	GH43	β-Xylosidase	Beta-xylosidase
GOI38	GH61	Polysaccharide monooxygenase	Auxiliary activity
GOI39	–	Hydrophobic binding protein	Auxiliary activity
GOI40	–	Swollenin	Auxiliary activity
GOI41	–	Hydrophobic binding protein	Auxiliary activity
GOI42	–	Hydrophobic binding protein	Auxiliary activity
GOI43	–	Swollenin	Auxiliary activity
GOI44	GH28	Polygalacturonase	Pectinase
GOI45	GH53	Galactanase	Pectinase
GOI46	–	Pectin lyase	Pectinase
GOI47	GH28	Endorhamnogalacturonase	Pectinase
GOI48	GH28	Endopolygalacturonase	Pectinase
GOI49	GH28	Exopolygalacturonase	Pectinase
GOI50	GH28	Exopolygalacturonase	Pectinase
GOI51	GH28	Xylogalacturonan hydrolase	Pectinase
GOI52	GH78	α-l-rhamnosidase	Pectinase
GOI53	GH78	α-l-rhamnosidase	Pectinase
GOI54	GH27	α-Galactosidase	Other side chain hydrolase
GOI55	GH67	α-Glucuronidase	Other side chain hydrolase
GOI56	GH67	α-Glucuronidase	Other side chain hydrolase
GOI57	–	Acetylxylan esterase	Other side chain hydrolase
GOI58	–	Acetylxylan esterase	Other side chain hydrolase
GOI59	–	Carboxylesterase	Other side chain hydrolase
GOI60	–	Carboxylesterase	Other side chain hydrolase
GOI61	–	Pectinesterase	Other side chain hydrolase
GOI62	–	Acetylxylan esterase	Other side chain hydrolase

The 700-bp upstream regions of the coding sequences of these GOIs were scanned for the canonical XlnR binding motif -5’-GGCTAA-3′- [55, 60, 61], as well as for the functional variant, low affinity XlnR binding motif 5′-GGCWAW-3′ [60]. We also searched for the CreA binding motif -5′-SCGGRG-3′- [62, 63]. It is worth noticing that almost all selected GOIs exhibit such regulatory sequences in their promoter: 85% possess at least one and up to five CreA motif, 80% present one to four XlnR motif, exactly half of them harbouring the canonical -5′-GGCTAA-3′ motif (Additional file 1). From the exhaustive list of the 170 putative genes encoding GH found in the annotated genome of *T. versatilis* (unpublished data), we found that these XlnR and CreA binding motifs present an interesting position bias in the promoter, half of the canonical XlnR binding sites (i.e. interquartile range) being located between − 190 and − 450 bp upstream the ATG, and between − 230 and − 480 bp for the CreA motif. These data are in agreement with those reported in *Fusarium* species for which the transcription binding sites mostly located within 600 bp from gene starts [64]. If their mere presence in the promoter regions does not necessarily result in their functionality with respect to transcriptional regulation (see discussion section), this observation suggests that some of these binding sites might be functional in *T. versatilis*.

### A global transcriptional landscape that highlighted the wide plasticity of gene responses, specific of ‘gene-condition’ pairs

**Fig. 2:**
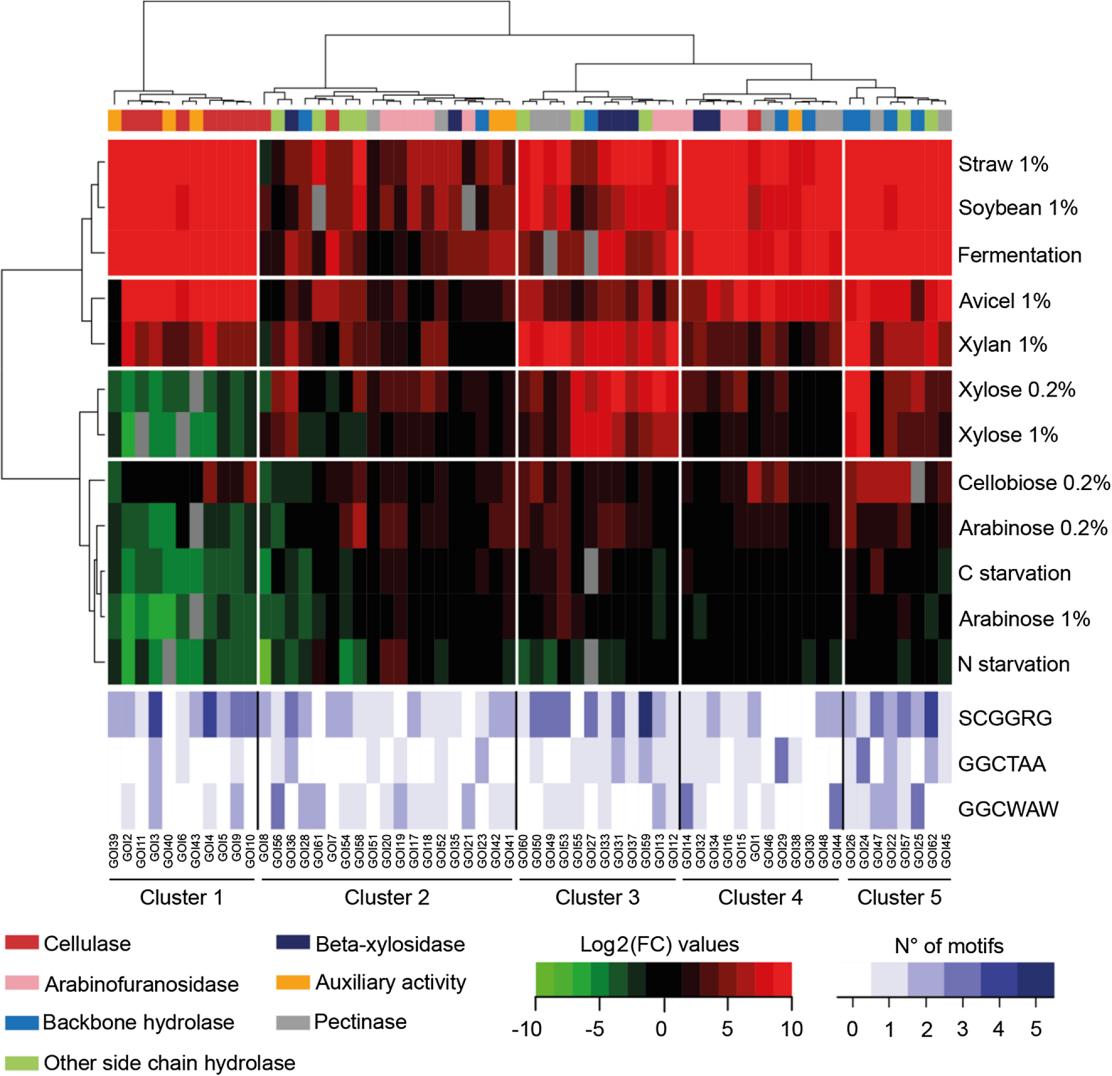
Transcriptional landscape in the WT during the shift from glucose to a condition of interest. Double hierarchical clustering of normalized fold change (FC) values, calculated using glucose as the calibrator sample, and expressed as log_2_ (FC). Clustered GOIs (top dendogram) and culture conditions (left dendogram), with the corresponding names at the bottom and right of the heatmap, respectively. The white lines in the heat map delineate the main clusters, i.e. 5 and 4 clusters for GOIs and culture conditions, respectively. The coloured bar at the top of the heat map is referring to functional categories affiliation of GOIs (legend at the bottom left of the figure). Red and green colours indicate increased and decreased FC values, respectively (scale at the bottom of the figure, with saturating intensity for absolute values of log_2_ (FC) beyond 9). Cq values that exceeded 35 cycles were considered beyond the limit of detection and represented by the grey cases in the heat map. The bottom, light blue heat map depicts the number of each of the 3 binding motifs identified in the 700 bp upstream the ATG start codon of each gene, i.e. the XlnR binding motif -5′-GGCTAA-3′, its low affinity variant 5′-GGCWAW-3′, and the CreA binding motif -5′-SCGGRG-3′

We explored 13 different nutritional conditions from simple sugars to complex lignocellulosic material, i.e. more or less heterogeneous polymeric substrates, including also carbon and nitrogen starvation. Normalized fold-change (FC) values of the 62 GOIs during the shift from glucose to the condition of interest, were calculated using glucose as the calibrator condition. The two-dimension hierarchical clustering presented in Fig. 2 identified five clusters of genes (horizontal axis), and four clusters of nutritional conditions (vertical axis) (see also Additional file 2: Figure S1 for quantitative visualisation of gene responses in the five clusters). Gene cluster 1 collected most of the genes encoding cellulases as well as few proteins with auxiliary activities. These genes were strongly induced in media containing carbon sources as straw (mainly hemicellulose), or avicel (mainly cellulose), with average log_2_ (FC) values higher than 10 (see also grey boxes in Additional file 3: Figure S2a). Conversely, they were downregulated by simple C5-sugars including xylose, as well as by carbon and nitrogen starvation, with an average 4-fold drop (log_2_ (FC)) as compared to glucose. The downregulation observed in xylose hence contrasted with their activation by xylan, which indicates that residues released by xylan can trigger induction signals that prevail over the downregulation due to the sole presence of xylose. A quite different situation was observed for cellobiose, a disaccharide made of glucose and arising from cellulose degradation, which led to a slight activation of most of the genes from this gene cluster 1 (average log_2_ (FC) of *approx*. 2 as compared to glucose, see Additional file 3: Figure S2a).

As for gene cluster 1, expression of GOIs from gene clusters 4 and 5 was remarkably upregulated in culture media containing polysaccharides (see also Additional file 3: Figure S2d, e), but they did not exhibit any downregulation in the other conditions. A specificity of cluster 5 genes was their better activation in the presence of xylan and xylose, particularly for the two xylanase encoding genes, *xynD* (GOI 24) and *xynF* (GOI 26). A good activation by xylan and xylose was also the most clear hallmark of gene cluster 3 (4 < log_2_ (FC) < 10, see Additional file 3: Figure S2c). The induction on xylose was however less potent than that observed in response to xylan, specifically for pectinases (see Additional file 4: Figure S3g), which indicates that xylose may act synergistically with other side-products of xylan hydrolysis for the induction of this functional category of genes, as previously indicated for cellulases. The remaining gene cluster, (number 2), which collected the largest set of GOIs and encoded enzymes with a wide spectrum of predicted hydrolytic activities, appeared quite insensitive to environmental stimuli when referring to this heatmap. This may come from the comparison with other gene clusters, which showed extreme responses to some culture conditions. However, the average expression of gene cluster 2 on straw, soybean and during fermentation was far from insignificant (log_2_ (FC) around 4, see Additional file 3: Figure S2b). Notably, polymeric substrates as well as xylose 0.2% sporadically gave rise to high FC values for a few genes (log_2_ (FC) > 5).

When referring to nutritional conditions, this 2D-cluster analysis allowed us to distinguish clusters of two distinct types (Fig. 2). A first type included complex lignocellulosic-based substrates that overall caused strong upregulation of almost all the GOIs (first and second nutritional clusters, on top of the heat map). The second type included simpler carbon sources, as well as carbon and nitrogen starvation, with a transcriptional response mainly characterized by the downregulation of cluster 1 genes. The singular positioning of the cellobiose condition, which clustered in this group while it did not lead to downregulation of cellulases, likely resulted from its overall modest activation of genes from clusters 2 to 5, similar to the activation observed on arabinose and xylose. Xylose also distinguished from these conditions, being able to trigger the huge activation of a subset of genes, mainly from gene cluster 3. Interestingly, xylose and arabinose showed a concentration-dependent response, as indicated by lower FC values of few GOIs as the concentration of these sugars increased from 0.2 to 1%, an effect that was particularly prevalent for genes encoding arabinofuranosidases and side chain hydolases (see Additional file 4: Figure S3c, d).

**Fig. 3:**
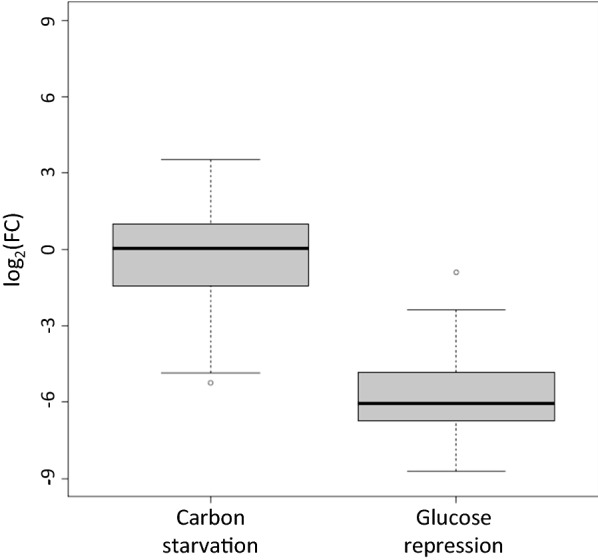
Focus on FC values during carbon starvation and after glucose addition. ‘Carbon starvation’: Shift of the mycelium from glucose medium to medium without carbon source (glucose condition used as calibrator sample; RT-qPCR data from this study); ‘Glucose repression’: addition of glucose to mycelium grown on wheat straw for 24 h (wheat straw condition as calibrator sample; RNA-seq data from a previous study [59]). The box-plot gathers log_2_ (FC) values of the 62 GOIs (i.e. those presented in the ‘C starvation’ line in Fig. 2, and those reminded in the Additional file 1 for the ‘glucose repression’ condition)

About the specific effect of carbon starvation, the left boxplot of Fig. 3 confirmed that the expression level of the overall set of GOIs did not significantly change upon removal of this carbon source from the medium, with the exception of the downregulation observed for cluster 1 genes. Conversely, this nutritional starvation moderately induced just a few genes distributed among gene clusters 2 to 5, mainly those encoding pectinases (see Additional file 4: Figure S3g). This phenomenon was therefore too marginal to conclude on a massive release of glucose repression that might have led to more generalized upregulation, as for example in *A. niger* [50]. Glucose repression was nevertheless an active mechanism in *T. versatilis*, as indicated by the strong repression that was observed upon glucose addition to *T. versatilis* mycelium grown on straw for 24 h (right boxplot in Fig. 3; data from our previous RNA-seq study [59]). In this later experiment, we indeed showed a massive extinction of total RPKM corresponding to GH, as it was previously shown in *A. niger* [65] and *T. reesei* [66] by using the same experimental strategy. To conclude, this modest activation of most of the genes in response to simple sugars, (i.e. arabinose or cellobiose, and even xylose, in the absence of glucose), suggested that best induction of most of these GOIs on hemicellulose- and cellulose-based substrates rely on the synergistic action of inducers that come from their hydrolysis.

### The loss-of-function of *xlnR* revealed a dual activator-repressor role of this transcription factor

**Fig. 4:**
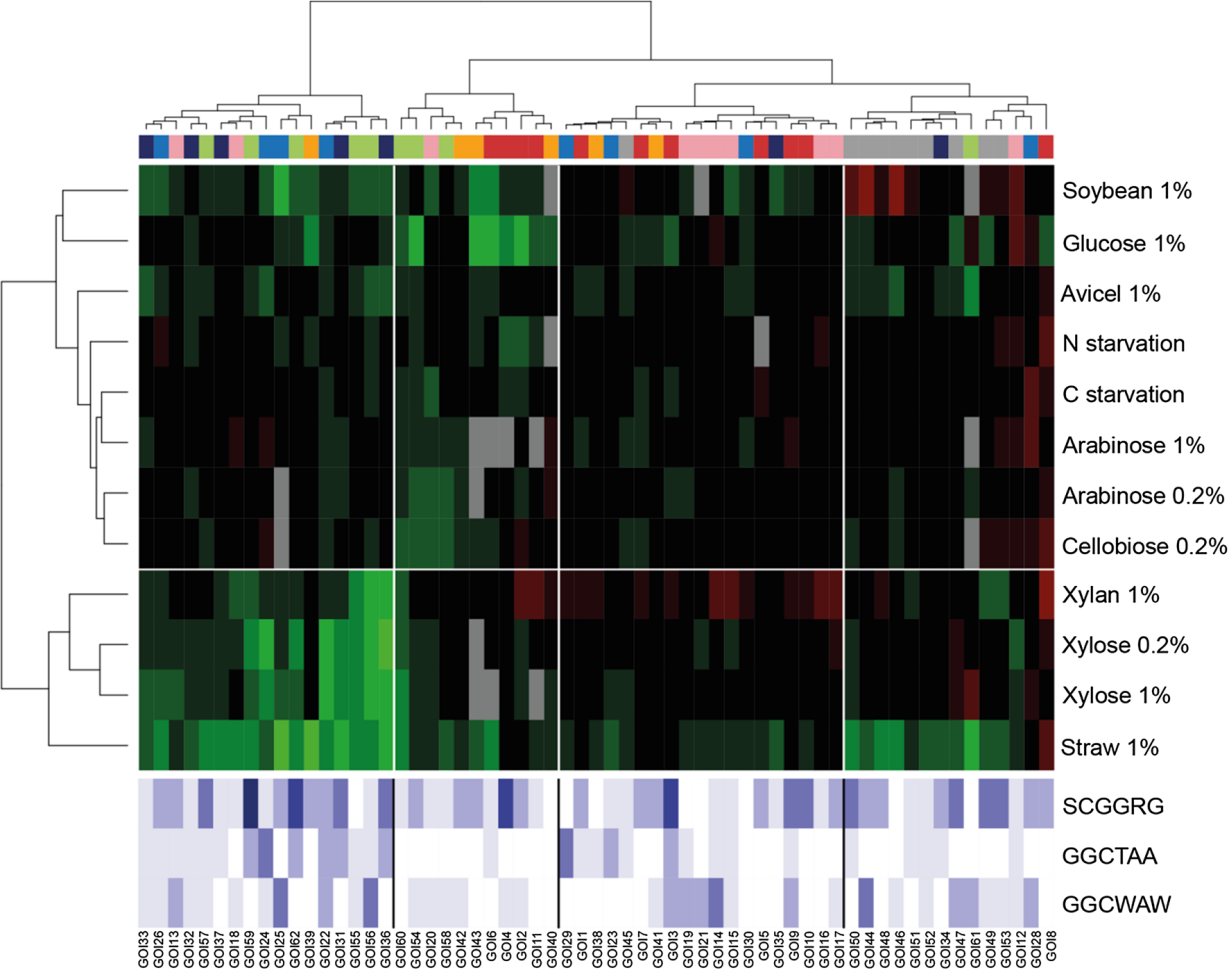
Remodelling of transcriptional patterns in the *ΔxlnR* mutant. Double hierarchical clustering of normalized fold change (FC) values between the *ΔxlnR* mutant and the wild-type strain, for each condition of interest (e.g. straw) (WT as the calibrator sample). We do not consider the shift from glucose to the various media for this calculation, the glucose condition being itself a condition of interest as the others, not a calibrator sample. Same legend as in Fig. 2

**Fig. 5:**
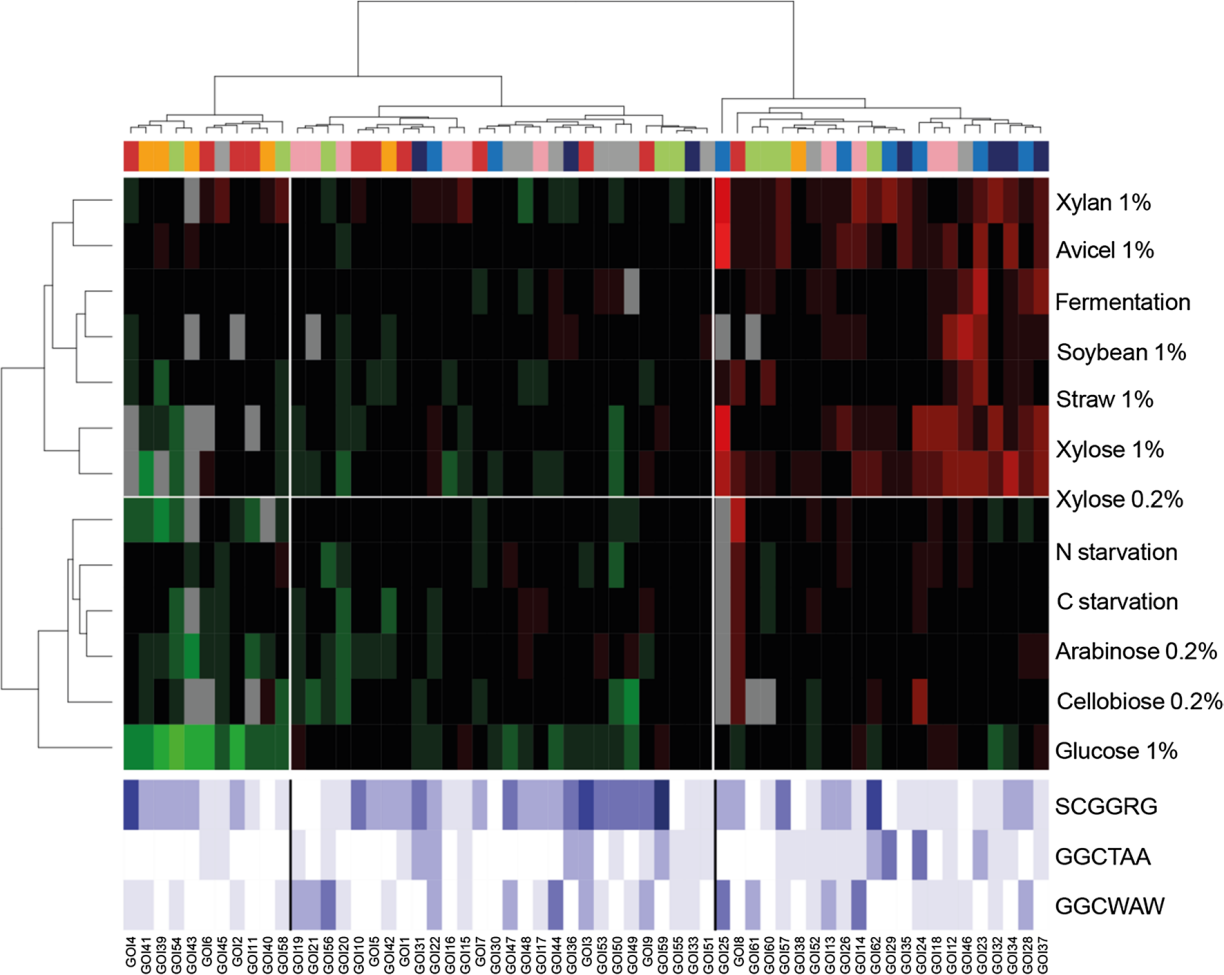
Remodelling of transcriptional patterns in the *xlnR*^+^mutant. Double hierarchical clustering of normalized fold change (FC) values between the *xlnR*^+^mutant and the wild-type strain, for each condition of interest (WT as the calibrator sample). We do not consider the shift from glucose to the various media for this calculation, the glucose condition being itself a condition of interest as the others, not a calibrator sample. Same legend as in Fig. 2

We previously identified in *T. versatilis* genome a putative homologue to *A. niger xlnR*, (62% identity at the protein level), and successfully deleted this gene [67]. The loss-of-function of *xlnR* in *T. versatilis* slightly reduced growth rate of the mycelium on PDA plates and in liquid MM (data not shown), but it did not abolish growth on the different carbon sources of the study, including xylan and xylose, contrary to what was observed in other fungi as *N. crassa*, *A. oryzae* and *Fusarium* species [61, 68–70]. In Fig. 4, FC values illustrate the ratio of normalised transcript levels between the mutant and the wild-type strain, in a condition of interest, and do not consider the shift from glucose to this medium. The glucose condition was therefore a condition of interest as the others, not a calibrator sample as in Fig. 2. Also, to avoid any ambiguity when referring to clustering results in the remaining part of the manuscript, even for description of classification results presented in the upcoming Figs. 4 and 5, we will solely give the name ‘gene clusters 1-5′ to the clusters that have been presented in Fig. 2 for the WT response.

The clearest hallmark upon deletion of *xlnR* was the reduced transcript levels for a subset of genes, which clustered in the left of the heatmap (Fig. 4). This subset was enriched for genes encoding xylanases, β-xylosidases and side chain hydrolases, in response to xylose, xylan and straw (bottom-left corner, Fig. 4). Very interestingly, while tracking the origin of these genes relative to their initial classification in the WT strain exposed to these carbon sources, almost all downregulated genes in the *ΔxlnR* background belonged to gene clusters 3 and 5 (See Additional file 3: Figure S2, and illustrative scatter plots in Additional file 5: Figure S4 for xylose, xylan and straw conditions). This observation was consistent with their strong activation when shifting the WT mycelium from glucose to xylose or xylan, and suggested a role of this TF in their activation. It has been stated that there is a core set of genes whose expression always depends on XlnR/XYR1, i.e. the genes coding for XlnD, XynB, XynA, BglA and AguA [57]. We identified the putative homologues of some of these genes in *T. versatilis,* as GOI 36 for XlnD, GOI 22 for XynB, GOI 55 and GOI 56 for Agu*A.* These genes also showed in *T. versatilis* a significant fall of their transcript levels in the absence of *xlnR*, both on xylose and xylan (5.5-fold drop (log_2_ (FC)), sub-cluster on the right of the bottom-left cluster in Fig. 4).

A noticeable observation for the xylan condition, not for xylose, was a counter-intuitive increase in the transcript levels of many GOIs (Fig. 4). These upregulated genes in the mutant strain mainly corresponded to gene clusters 1 and 4 in the WT strain (Additional file 3: Figure S2a, d), and more precisely to genes encoding cellulases and most arabinofuranosidases (Additional file 4: Figure S3c, f). These data indicated that XlnR might have, on xylan specifically, a dual activator-repressor role. This was consistent with the results observed in the WT strain exposed to this carbon source, as we observed in Fig. 2 a much better xylan-dependent activation of GOIs from gene clusters 3 and 5, positively regulated, than GOIs from gene clusters 1 and 4, negatively regulated by XlnR. Interestingly, these negatively regulated GOIs on xylan were conversely positively regulated during growth on xylose, as well as on other polymeric carbon sources as straw. We can therefore propose that in the wild type strain grown on xylan, the repressor function of XlnR on the transcription of most cellulases and arabinofuranosidases is triggered by specific side-products of xylan hydrolysis, others than xylose, and not present in other polymeric substrates.

Another remarkable feature of *ΔxlnR* cells was the general downregulation observed on straw, more pronounced than in response to xylose, both for the amplitude of the drop of transcript levels, and for the number of GOIs affected by the deletion (Additional file 5: Figure S4c). A similar, generalized effect was observed to some extent on other carbon sources such as avicel and soybean. However, for *ΔxlnR* cells exposed to soybean, a notable hallmark was the upregulation of GOIs (right gene cluster in Fig. 4). This cluster gathered all pectinases except GOI45, yet similarly upregulated on soybean. This specific, soybean-triggered upregulation of pectinases in the *ΔxlnR* strain, contrasted with their significant downexpression in the presence of other carbon sources as straw and avicel (see also Additional file 4: Figure S3g). As previously mentioned for xylan, this feature highlighted another example of dual activator-repressor role of this transcription factor, whose gene-targets are strictly dependent on the nature of the carbon source.

The sole presence of glucose also appeared as a favorable nutritional context to observe a *ΔxlnR*-dependent, generalized down-regulation of GOIs, with at least 3-fold drop of transcript levels for 25 GOIs. These downregulated genes on glucose encoded, amongst others, 6 out of the 11 cellulases, 5 out of the 10 pectinases, and 4 out of the 6 proteins with auxiliary activities. On other simple carbon sources, the deletion of *xlnR* did not affect the expression of genes significantly, with the notable exception of genes encoding side chain hydrolases on cellobiose (see Additional file 4: Figure S3d).

### The multicopy integration of *xlnR* surprisingly boosted transcript levels of very limited subsets of genes

In the wild type strain, we could notice moderate two- to five-fold activation of *xlnR* itself during the shift from glucose to the different nutritional conditions (data not shown), which may participate in transcription responses depicted in Fig. 2. It has been reported that addition of extra copies of the xylanolytic regulator or its overexpression can increase transcription of glycosyl hydrolases encoding genes, such as xylanases and endoglucanases, with parallel increase of their secretion [69, 71–75]. We therefore investigated the effect of *xlnR* overexpression in *T.* *versatilis* by using a strain that carries 9 copies of *xlnR* under the control of its own promoter (*xlnR*^+^ strain), as determined by a digital droplet PCR assay on genomic DNA. As in the previous section, the FC values of GOIs were calculated as the ratio of normalised transcript levels between the *xlnR*^+^ and the wild-type strain, in each condition. About *xlnR* transcript levels themselves (data not shown), an *approx*. 8-fold increase of transcript levels could be observed in the *xlnR*^+^ strain incubated on xylose and xylan, which correlated to the copy number of *xlnR* in this strain. Its overexpression was still significant for the remaining polymeric substrates as for cellobiose (FC around 6), and less pronounced for arabinose, starvation conditions and glucose (2 < FC < 4), confirming the strong dependency of transcript levels upon nature of the carbon source.

Not surprisingly, data presented in Fig. 5 showed that the consequence of *xlnR* overexpression was also highly dependent upon nutritional environment. Globally, it affected the expression of *approx*. one-third of GOIs (upper-right cluster), clearly enriched for genes encoding hemicellulases. We observed this positive transcriptional effect under xylose and complex substrates, not on arabinose and starvation, neither on cellobiose while this latter carbon source led to a clear accumulation of *xlnR* transcripts in this *xlnR*^+^ strain. In more details, the best substrates to promote the effect of *xlnR* overexpression were xylan and xylose (26 and 20 GOIs with FC > 3, (i.e. log_2_ (FC) > 1.58), respectively, see also Additional file 6: Figure S5a, b), and to a lesser extent, avicel (15 GOIs), fermentation (14 GOIs, see the discussion section) and soybean (10 GOIs). Much to our surprise with respect to *xlnR* transcript levels themselves, straw was the least efficient condition of this group, as just a few GOIs showed a positive response to *xlnR* overexpression (only 7 GOIs with FC > 3, see also Additional file 6: Figure S5c). As is shown in Additional files 3: Figure S2 and 4: Figure S3 to better track these genes, xylan and xylose mainly triggered overexpression of GOIs originating from gene clusters 4 and 5, and particularly genes encoding hemicellulases (i.e. beta-xylosidases, backbone hydrolases, and to a lesser extent, side chain hydrolases and arabinofuranosidases). On the contrary, a relevant hallmark was the insensitivity to *xlnR* overexpression of GOIs originating from gene cluster 1, i.e. mainly cellulases-encoding genes, whatever the condition (see Additional file 4: Figure S3f).

**Fig. 6:**
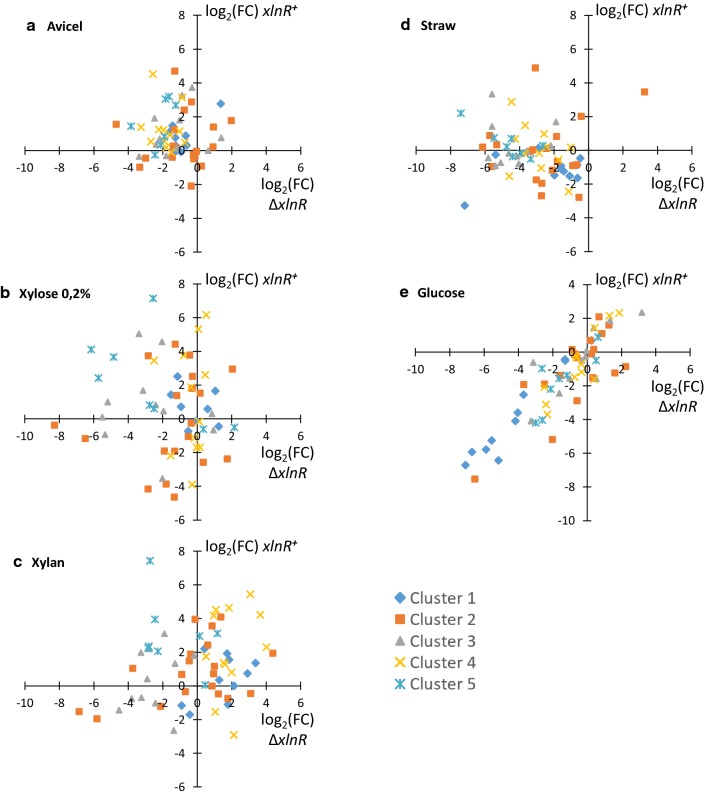
Specificities of *xlnR*^+^ and *ΔxlnR* responses under different culture conditions. Scatter plots of log_2_ (FC) values in the *xlnR*^+^ strain as a function of log_2_ (FC) values in the *ΔxlnR* strain. One scatter plot per culture condition. The GOIs are gathered according to the five gene clusters defined in Fig. 1

As it was previously found with the unexpected *ΔxlnR*–triggered upregulation of cellulases on xylan, and of pectinases on soybean, *xlnR*^+^ strain on xylose highlighted a similar dual repressor-activator function of this TF. It was illustrated with the unforeseen downregulation of a dozen of genes showing FC values lower than minus 3, particularly half of the genes encoding arabinofuranosidases, (6 out of the 10 from this category), as well as side chain hydrolases and proteins with auxiliary activities (Additional file 4: Figure S3). The most unsettling result came from the glucose condition, which also led to a massive downregulation of GOIs in the *xlnR*^+^ strain, (22 genes with FC ≤ 3, see below and Fig. 6e), particularly those encoding cellulases and proteins with auxiliary activities. A similar trend was observed for this specific set of GOIs on cellobiose, with their downregulation. These results emphasized that the possibility for XlnR TF to boost -or repress- target GOIs, is highly dependent upon the nature of the carbon source and on mechanisms other than the regulation of the sole XlnR protein content. This was illustrated for example by avicel and cellobiose conditions, leading to similar *xlnR* changes both in the WT and in the *xlnR*^+^ strain, yet showing highly divergent expression patterns of GOIs, with upregulation of hemicellulases for the former and downregulation of cellulases and auxiliary activities for the latter.

### *xlnR*^+^ versus *ΔxlnR* responses: no generalizable shape, only substrate-specific patterns reflecting the complexity of XlnR-dependent transcriptional regulation

When considering the genes most affected by the overexpression of *xlnR* (i.e. upregulated genes of the top-right cluster in Fig. 5) or by its deletion (i.e. downregulated genes of the bottom-left cluster in Fig. 4), we found that among the 30 collected genes, only 9 were affected both by *xlnR* deletion and by its overexpression. More focused, condition-specific analysis nuanced this conclusion, as illustrated in Fig. 6 that plots log_2_ (FC) ratios in the *xlnR*^+^ strain as a function of the *ΔxlnR* strain, where genes expected to be downregulated in the *ΔxlnR* strain and upregulated in the *xlnR*^+^ strain, will locate in the upper-left sector of these scatter plots. The most pertinent pattern was obtained for strains grown on avicel (Fig. 6a). On this carbon source, downregulation observed in the *ΔxlnR* strain was not strong (− 3 < log_2_ (FC) ≤ 1 for most of GOIs), yet massive. Reciprocally, avicel was also the most efficient carbon source to trigger quite generalized upregulation of gene expression in the *xlnR*^+^ strain, leading to positioning of most of GOIs in the upper-left sector of the scatter plot.

Consistently with results pointing to XlnR being a dual repressor-activator TF, more composite distributions were observed in xylose and xylan scatter plots. On xylose (Fig. 6b), among the 21 hemicellulases that were downregulated in the *ΔxlnR* strain (FC ≤ 3), only 9 were also upregulated in the *xlnR*^+^ strain (FC > 3), with an enrichment of backbone hydrolases originating from gene cluster 5. The remaining 12 genes were either weakly upregulated and not considered, or, in agreement with the repressor role of XlnR, downregulated in the *xlnR*^+^ strain as were most of arabinofuranosidases. On xylan (Fig. 6c), as on xylose, few genes encoding hemicellulases originating from gene cluster 5 and few others from cluster 3 showed the expected expression pattern, i.e. downregulation in the *ΔxlnR* strain and upregulation in the *xlnR*^+^ strain. But this behaviour was marginal, as many upregulated genes in the *xlnR*^+^ strain were also upregulated in the *ΔxlnR* mutant, leading to a massive positioning of the genes in the upper-right sector of the graph, particularly for those encoding arabinofuranosidases and originating from gene cluster 4. Another remarkable situation was observed on straw (Fig. 6d), with very few GOIs responding positively to *xlnR* overexpression while this substrate was the most relevant to observe a massive, downregulation of the full set of GOIs in the *ΔxlnR* mutant. Taking into account the data presented in Additional file 4: Figure S3g, we can speculate that the higher the expression in the WT strain, as on straw, the lower the possibility to further promote transcript levels under *xlnR* overexpression.

Finally, the most outstanding pattern was observed when analyzing the effect of XlnR transcription factor on glucose (Fig. 6e), with almost all the genes being downregulated in both strain. While tracking the identity of the genes, this behavior particularly affected categories as cellulases, (6 out of the 11 GOIs with FC ≤ 3, four others being nevertheless downregulated in both strain), pectinases, (5 out of the 10 GOIs with FC ≤ 3, two others being also downregulated in both mutant), and enzymes with auxiliary activities (all the 6 members, 4 significantly affected with FC ≤ 3).

### To belong to the same functional category did not necessarily translate into similar regulatory patterns

**Fig. 7:**
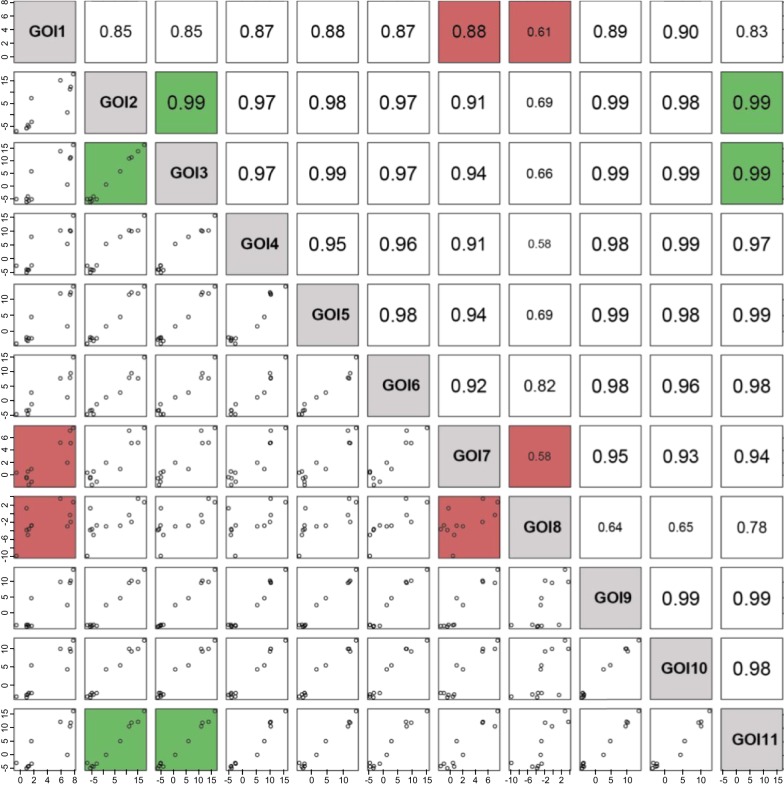
Pairwise correlations between genes encoding cellulases, in the WT strain. GOIs’ labels are given in the diagonal of the matrix (grey squares). Bottom-left part of the matrix: Scatter plots of log_2_ (FC) values between two genes (Fig. 2 data, WT strain). Log_2_ (FC) values’ scale is reported on the bottom and left edge of the matrix, for each scatter plot (x- and y-axis, respectively); Upper-right part of the matrix: Pearson correlation coefficient (*r*) between these two genes (the higher the police size, the better the coefficient). We arbitrary highlighted in green the three GOIs that clustered in the very lelft of the dendrogram (cluster 1), and in red, three divergent ones found in gene clusters 2 and 3

**Fig. 8:**
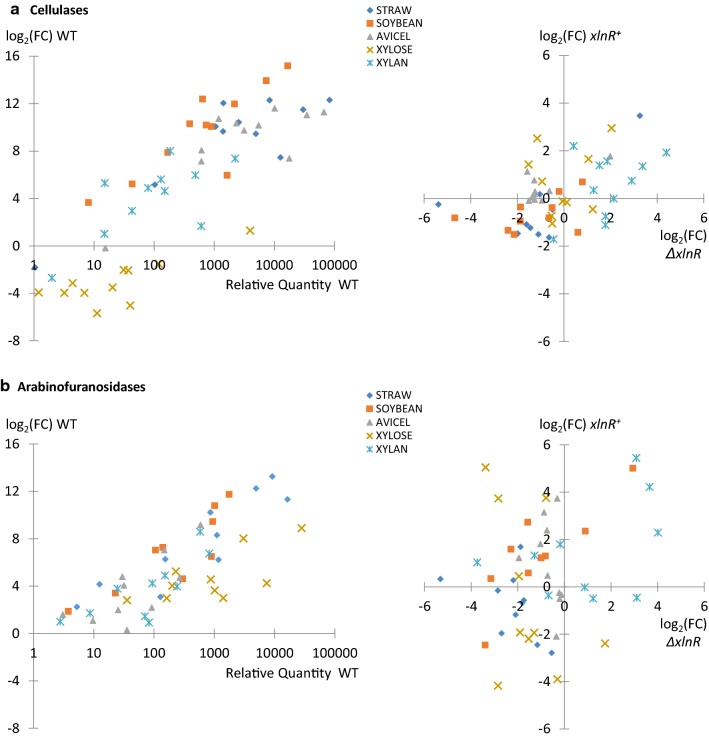
Heterogeneity of expression data within functional categories. **a** 11 GOIs encoding cellulases and **b** 10 genes encoding arabinofuranosidases, in response to five selected carbon sources (coloured code). Left scatter plot: log_2_ (FC) values, (i.e. shift from glucose to the medium of interest as in Fig. 2), as a function of the relative quantities of transcript levels (RQs), in the wild type strain. Right scatter plot: log_2_ (FC) values in the *xlnR*^+^ strain as a function of log_2_ (FC) values in the *ΔxlnR* strain. RQs were calculated using as reference the ‘GOI-condition’ pair sample leading to the highest Cq value in the full set of data from this functional category of genes (i.e. lower transcript level). No symbol drawn at RQ of 1 for the arabinofuranosidases category (**b**), as the culture condition leading to the lowest expression within this group of genes was not plotted (i.e. 5 conditions plotted amongst the 12 used for calculation)

Data that have been presented in Fig. 2 first illustrated that members of a functional category were generally widespread within the different clusters of the heatmap, revealing the heterogeneity of their response to the different nutritional conditions. Genes encoding cellulases appeared as an exception as almost all members gathered in gene cluster 1. Going into details however, three additional genes encoding cellulases showed some divergence in their overall response. GOI 8 (cluster 2) was not induced by the polymeric carbon sources, while GOIs 7 and 1 (cluster 2 and 4, respectively) were unaffected by the overall downregulation of cluster 1 genes. Similarities and differences between cellulases were best illustrated by pairwise comparisons presented in Fig. 7. We could observe almost perfectly correlated genes (e.g. GOIs 2, 3 and 11; Pearson correlation coefficient of 0.99), and more divergent ones (e.g. GOIs 7, 8 and 1; 0.58 < *r* < 0.88). Second, the relative quantity of transcript levels appeared as another type of heterogeneity, even in the WT strain (Fig. 8a, x-axis of the left scatter plot). Similar FC values during the shift from glucose to a condition of interest (y-axis) could indeed hide significant differences in the strength of their promotor (3- to 4-log). Third, the scattering of the genes in response to the different *xlnR* backgrounds, previously discerned in Figs. 4 and 5 heatmaps, was perfectly illustrated when plotting log_2_ (FC) ratios in the *xlnR*^+^ strain as a function of the *ΔxlnR* strain (Fig. 8a right scatter plot). Whether for xylan, xylose or soybean, both the type of regulation (up- or downregulation) and the amplitude of FC values varied significantly from one gene to another. For substrates as straw or avicel, a better clustering of most of the members did not prevent the presence of outliers, away from the median response of this functional category.

The heterogeneity of responses in the regulation of the arabinofuranosidase category was another interesting example. As compared to cellulases, we could notice wider differences between members, both for FC values and relative quantities of transcript levels in a condition of interest (Fig. 8b, left), and for the singularity of patterns in the different *xlnR* backgrounds that clearly illustrated the dual activator-repressor function of XlnR (Fig. 8b, right). Finally, we examined coregulations between genes encoding backbone hydrolases when changing *xlnR* genetic background, as we previously underlined that a few members of this functional category fitted with logic down- and upregulation in the *ΔxlnR* and *xlnR*^+^ strains, respectively, especially on xylose. The correlation matrices drawn for the WT, *ΔxlnR* and *xlnR*^+^ strains (Additional file 7: Figure S6), showed that even for the few genes that exhibited rather good pairwise correlations in the WT strain (Additional file 7: Figure S6a, arbitrary green-coloured squares when *r *> 0.90), these correlations were dismissed in the mutant strains. This was illustrated by the clear dispersion of the points and drop of Pearson’s coefficients in the *ΔxlnR* and *xlnR*^+^ matrices (refer to the same, green-coloured squares in Additional file 7: Figure S6b, c). This result indicated that coinduction of two genes in the WT strain, under the different substrates, did not necessarily go along with quantitative coregulation of these genes under *ΔxlnR* and *xlnR*^+^ backgrounds. As a whole, all these observations showed that no consensus response could be proposed within a functional category.

## Discussion

### A complex transcriptional landscape, mainly shaped by medium composition

In this work, we have analysed the expression patterns of 62 representative genes encoding hydrolytic enzymes in the fungus *Talaromyces versatilis*, (formerly known as *Penicillium funiculosum*), to highlight key features in their transcriptomic response to different carbon sources. As previously reported in different fungi [70, 76–81], our study underlined the huge plasticity of transcriptional responses when changing nutritional status, with complex patterns of co-regulated groups of genes. As a general trend, the more heterogeneous the polymeric substrate, the more efficient to trigger transcriptional activation when *T.* *versatilis* was shifted from glucose to these rich media, whose biochemical composition further changes dynamically during its assimilation [50]. Especially, growth on wheat straw, or on the fermentation medium used for industrial cocktail production, led to a powerful activation of nearly all GOIs as previously described for *A. niger* and *T. reesei* also grown on wheat straw [65, 66, 78]. However, despite global similarity of the responses to polymeric substrates, differences in the expression levels of individual genes were observed, as it was found in *A. niger* after comparative RNA-seq based transcriptome responses to wheat straw [65] and coppice willow [79].

More simple sugars as arabinose and cellobiose appeared as weak inducers in *T. versatilis*, strengthening the idea that efficient inducing mechanisms also rely in this fungus on the production of small molecular weight compounds that act in a synergistic manner [34, 50, 65]. Another illustrative result was the comparison of pure xylose versus xylan as substrates: these two carbon sources upregulated almost the same set of genes but the induction was less effective on xylose than on xylan, a result that was already stated by van Peij et al. [72]. In our study, this effect was particularly marked for pectinases, indicating that side-products of xylan hydrolysis may act synergistically with xylose to promote expression of these genes. Similarly in *A. niger*, wheat straw pretreatment abolished the expression of a large set of genes encoding pectinolytic enzymes [82]; these authors explained their observation by the removal of parts of the lignocellulose during pretreatment of this substrate, components which were probably important for induction.

### A limited role of XlnR as direct, positive regulator

The transcription factor XlnR/XYR1 [55], appeared over time as a key regulator for activation of both hemicellulolytic and cellulolytic genes. Expression of most *xlnR*/*xyr1*/*xlr1* orthologs is not specifically induced (reviewed in [53]), with a few exceptions as in *T. reesei* where *xyr1* expression appeared strongly activated by sophorose, lactose and cellulose [83, 84]. It was also reported that addition of extra copies of this TF or its overexpression can increase transcription of target genes, with parallel increase of enzyme secretion [69, 71–75]. Again, counterexamples could be found, with the report of a limited impact of this overexpression [85, 86].

As *T. versatilis* genome encodes a protein with strong homology to *A. niger* XlnR/XYR1, we therefore analysed the effects of both the deletion and the overexpression of *xlnR* in this fungus. In our study, we could notice moderate two- to five-fold activation of *xlnR* itself during the shift from glucose to the different nutritional conditions (data not shown). Overexpression upon multicopy integration of *xlnR* further promoted its transcript levels, but overall, whether for the wild type or the overexpression strain, the relatively modest, carbon source-dependant changes in *xlnR* transcript levels themselves contrasted with the extreme transcript levels of some putative XlnR target genes. These results hence supported the idea that, as in other fungi, XlnR-dependent gene expression does not primarily rely on the sole control of its transcript levels, but on post-translational control [87].

Both mutations, either the deletion or the overexpression of *xlnR*, indeed led to significant reorganisation of GOIs’ transcriptional patterns, confirming its role in the global regulation of gene network in *T. versatilis*. However, the full set of “gene-condition” pair possibilities, in the three strain backgrounds, highlighted an outstanding level of complexity of coregulations, especially illustrated by the diversity of *xlnR*^+^ versus *ΔxlnR* patterns. Just a minimal set of GOIs and/or conditions actually fitted in a simplistic model of regulation by a transcriptional activator, i.e. downregulation in the *ΔxlnR* strain and upregulation under XlnR overexpression. The avicel was not the most powerful carbon source for transcriptional activation, but almost all GOIs respected this simple regulatory scheme. Xylose and xylan, classically reported as inducers of hemicellulolytic gene expression, actually triggered the expected down- or upregulation of a tiny group of genes from cluster 5, mainly encoding xylanases and beta-xylosidases. As these few genes showed an enrichment for the XlnR binding motif in their promoter region, (average of 2.5 motif/gene if considering both the canonical and low affinity motif, versus 1.5 for the whole selection of GOIs), these specific regulatory responses in the WT and mutant strains may rely on a positive and probably direct binding of XlnR on the promoter of these genes. Targeted ChIP-PCR or more global ChIPseq-based experiments as in [88] might nevertheless be necessary to clarify the specific role of XlnR and define directly regulated genes by this TF in these conditions, but also in all conditions of interest. Indeed, while variable number of genes were downregulated in the *ΔxlnR* mutant strain, the moderate and even the absence of effect of XlnR overexpression was conversely very surprising in conditions other than avicel and xylose. On straw for example, the massive, downregulation of the full set of GOIs in the *ΔxlnR* mutant indeed contrasted with the ineffectiveness of its overexpression. Such a lack of activation upon XlnR/XYR1 overexpression was also reported for transcriptional activation of xylanases in *F. oxysporum* [86] and might find explanation in the post-translational control of this TF. Specifically, it was proposed that already high and saturating activity of XlnR in the wild type strain, e.g. through various levels of phosphorylation elicited by such lignocellulolytic substrates [87], could prevent further activation of gene expression.

Beyond these mechanistic considerations and bearing in mind the zootechnical performance of the ‘Rovabio^®^ Advance’ cocktail that is produced from the *xlnR*^+^ strain, (e.g. body weight gain and feed conversion rate, personal communications from ADISSEO FRANCE SAS and [36]), we specifically focused on this surprising, limited number of overexpressed genes in the fermentation condition (i.e. industrial process). Indeed, solely 14 hits with FC values higher than 3 were identified in this strain, i.e. 5 out of 10 genes encoding pectinases and just a few genes encoding hemicellulases (2 backbone hydrolases and 2 beta-xylosidases, 3 arabinofuranosidases, 2 side chain hydrolases). Proteomic analysis of the secreted proteins by the wild type and the *xlnR*^+^ strain at the end of the fermentation process ([37] and personal communication from O. Guais, CINABio-ADISSEO), showed that despite comparable overall protein concentration and similar number of hits in the secretome of these strains, (67 and 65 characterized proteins, respectively), *xlnR*^+^ secretome was enriched in pectinases and hemicellulases. These results actually agree with overexpression data and reinforce the idea that targeting just a minimal number, yet adequate set of genes, can enhance the performance of a cocktail.

### Literature-based predictions of gene regulation: a risky strategy

The influence of the carbon source appeared of prime importance to delineate the set of regulated genes in the strains of interest. In that context, our and previous results from fungal literature showed that it definitely seems difficult to predict the effect of XlnR, even when referring to data from closely related fungi [57, 77, 81]. The plethora of conditions found in the reports, (e.g. nature of the substrate and/or inducer, duration and concentration of the exposure, strain background and overexpression strategy), altogether influence the role of the transcriptional regulators and ultimately, conclusions on gene responses. This makes comparisons between studies extremely tricky, and generalizations often biased, particularly when they are drawn irrespective of the carbon source, or based on a minimal number of GOIs and/or non-overlapping sets of genes between the studies.

A focus on genes encoding cellulases illustrates this diversity of regulatory mechanisms between media and/or fungi (see the review by [89]). For example, a statement as *T.* *reesei* being the only fungus with a clear XYR1 dependent regulation of the cellulolytic system must be nuanced, in light of observations made in *A. niger* [72] or *A.* *oryzae* [69] where their transcription was also heavily impaired or abolished. Other studies concluded for the absence or slight effect of XlnR/XYR1 system on cellulase production in *N.* *crassa* [70], *F. graminearum* [68] or *T.* *cellulolyticus* [90]; However, slightly different *T. cellulolyticus* strain backgrounds led to different conclusions between Fujii’ and Okuda’ studies [75, 90], XlnR being from the latter the main regulator for cellulase gene expression and activity in this fungus. Globally, Kunitake’s statement that the regulatory system of cellulolytic enzymes is not only partially conserved, but also significantly diverse [89], can probably be applied to most fungal systems. It also strengthens the idea that experimental reassessment of gene responses is mandatory if conclusions must be drawn in other experimental contexts, whether nutritional or genetic. Results obtained in *T.* *versatilis* did not contravene this idea.

Relative to other fungi, the global behaviour of genes encoding cellulases in *T.* *versatilis* appeared quite reminiscent of *N. crassa* [70], as they presented almost no effect of XlnR, whether for the deletion or for the overexpression of this TF. This overall behaviour also distinguished these genes from those encoding the other categories of enzymes, suggesting that XlnR should not be involved in the control of their expression in *T.* *versatilis*. This without counting on their unpredicted downregulation upon *xlnR* overexpression on cellobiose, and their upregulation upon *xlnR* deletion on xylan. Such indication for a repressor function of XlnR on cellulases has been reported in literature, yet on glucose, with the upregulation of many genes upon *xyr1* deletion in *T.* *reesei* [77]. In *T.* *versatilis* grown on glucose, we observed the reverse situation with almost all the genes encoding cellulases being clearly downregulated in the deletion strain (10 out of the 11 candidates), the most outstanding result being nevertheless, still on glucose, their similar downregulation upon *xlnR* overexpression. Mechanistically, this striking, downregulation in both mutant strain grown on glucose was inexplicable. But these effects of XlnR were most likely indirect, as a decrease of XlnR motif representation was observed in their promoter as compared to the whole set of GOIs (either the canonical or the low affinity consensus motif), which was actually consistent with the overall lack of effect of XlnR on these genes.

As a conclusion, if we can minimize the role of XlnR on cellulase regulation in our whole set of experimental conditions, the strong induction of these genes in the wild type strain grown in the presence of cellulolytic and hemicellulolytic substrates implies the existence of alternative transcriptional control. Together with their activation by cellobiose, yet modest, as in *N.* *crassa* [91, 92], all these results point to the possible contribution of CLR-2/ClrB ortholog, also found in *T.* *versatilis* genome and proposed as a major regulator of the cellulolytic system in filamentous fungi as *N. crassa, A. nidulans* and *A. aculeatus* [91, 93].

### The dual repressor-activator role of TFs: not so marginal observation in *T. versatilis*, and beyond

Besides genes encoding cellulases, this study revealed other examples of counter-intuitive transcripts regulations that targeted very specific sets of genes, and this, in precise nutritional contexts. Relevant situations were the lower expression of genes encoding auxiliary activities under XlnR overexpression, during growth on xylose and cellobiose; The upregulation of few genes encoding arabinofuranosidases in the *ΔxlnR* strain on xylan, and their downregulation in the *xlnR*^+^ strain on xylose; The upregulation of pectinolytic genes in the *ΔxlnR* strain on soybean, or their downregulation in the *xlnR*^+^ strain on arabinose. Such observations of a dual, substrate-dependant repressor-activator role of XlnR are not new or unusual. We previously cited the upregulation of many cellulase encoding genes upon *xyr1* deletion in *T. reesei* grown on glucose [77]; Bischof et al. highlighted a similar upregulation of gene expression in a *Δxyr1* mutant of *T.* *reesei*, for wheat-straw induced chitinases and mannosidases [94]. However, based on variable occurrence and even absence of the Xyr1 binding site in the promoter of these genes, these authors proposed that the action of Xyr1 as a repressor was very unlikely, and they rather considered an indirect, growth-related induction mechanism as this deletion mutant was unable to grow on wheat straw. This alternative seems unlikely in *T.* *versatilis* since this fungus did not exhibit such growth defects. In addition to XlnR, Kunitake et al. reported highly variable effect of ClbR overexpression on cellulolytic gene expression in *A. aculeatus* [95], with both up- and downregulated genes as compared to WT strain, which is quite reminiscent of the very heterogeneous behaviour of our set of arabinofuranosidases in the *xlnR*^+^ strain grown on xylose. Castro et al. also found that various genes encoding cellulolytic and xylanolytic enzymes were downregulated in the *T.* *reesei Δcre*-*1* mutant, even in presence of glucose, suggesting that CRE-1 can act positively on these genes in a direct or indirect manner [77]. Similar observations were reported by Sun et al. with a huge cluster of downregulated genes in the *N. crassa* *Δcre*-*1* mutant, demonstrating that CRE-1 functions as a global transcription factor affecting both gene repression and activation in *N. crassa*, both directly and indirectly [96]. Still in this fungus, Thieme et al. recently reported the de-repression of several pectinolytic genes in the absence of PDR-1 [97]. Several lines of evidences allowed them concluding that besides its activator role, this function in repression of transcription most likely occurs indirectly, in concert with one or several TFs as part of a larger regulatory network. All these examples of dual activator-repressor role of transcription factors, including XlnR, strengthen the extreme specificity of transcriptional responses, with strong influence of both the carbon source and the promoter region context on transcription factors properties.

### Glucose effects in the transcriptional control of GH: more complex patterns than for the expected CCR

The general mechanisms leading to Carbon Catabolite Repression (CCR) is largely conserved in *S. cerevisiae* and filamentous fungi during growth in the presence of glucose (Review by [54]). In filamentous fungi, this mechanism relies on the pivotal transcriptional regulator CreA/Cre1, whose putative homolog was also identified in *T.* *versatilis* genome. As it was previously shown in *A. niger* and *T. reesei* [65, 66], glucose did appear in *T.* *versatilis* as a formal repressive sugar if referring to the massive repression of most GOIs, including cellulases, upon glucose addition to the mycelium grown on wheat straw. While studying the role of PfMig1, (i.e. Cre1/CreA homolog), in the *Penicillium funiculosum* NCIM1228 strain, (also found as *Talaromyces funiculosus*), Randhawa et al. similarly described a PfMig1-dependant, repressive effect of glucose when this sugar was added to the fungus grown on avicel, with major drop of cellulase activity [98]. This later result suggests that in *T. versatilis*, the effect of glucose addition on transcript levels also relies on this transcription factor Cre1/CreA/Mig1. Besides glucose repression, we also noticed a dose-dependent inhibitory effect of xylose and arabinose when used as sole carbon sources. A similar effect was observed in other filamentous fungi, which do not only induce CCR in response to glucose but also to high concentrations of alternative carbon sources, as cellobiose and xylose in *A. niger*, *A. nidulans* and *T. reesei* [83, 99–102]. In such conditions of high substrate concentration, CreA/Cre1 predominantly localized in the nucleus, hence leading to transcriptional repression [83, 99, 102]. Such a mechanism should be transposable to *T. versatilis,* however, this dose-dependent inhibitory effect of xylose and arabinose was observed only for arabinofuranosidases and side chain hydrolases, not for xylanases and cellulases as in other fungi.

The massive drop of transcript levels of most GOIs upon addition of glucose could be considered as a definite proof that CCR is effective in *T. versatilis*. However, another interesting condition was the removal of glucose from the culture medium (i.e. glucose starvation condition), also found as “de-repressed” or “no-Carbon” condition in literature. The (large-scale) studies carried out in *T. reesei*, *A. niger*, *A. nidulans* and *N. crassa*, reported the release of repression of cellulolytic and xylanolytic genes in the absence of glucose [50, 63, 65, 76, 77, 91, 96, 99, 103], whose products may then act as scouting enzymes playing a foraging role on complex substrates, releasing the inducing molecules that trigger the main hydrolytic response [50, 103, 104]. With the exception of just a few derepressed genes, mainly encoding pectinases, no clear derepression has been observed in *T. versatilis* under glucose starvation condition. On the contrary, we surprisingly observed a drop of transcript levels for cluster 1 genes, (mainly cellulases), which contrasted with previous reports showing *cbh1* expression being unchanged [76, 105] or moderately activated [77] during the shift of *T. reesei* from glucose to a starvation condition. As this phenomenon was also observed with simple, alternative sugars as xylose and arabinose, we favoured the idea that these lower transcript levels of genes encoding cellulases actually result from the absence of glucose after the shift to these different media. That is indeed a common denominator in these conditions, with the exception of the N-starvation context that contained glucose but showed moderate downexpression of almost all GOIs in the WT strain. To our knowledge, this is the first report of a negative effect of the absence of glucose on such functional categories of genes, (here those encoding cellulases and most auxiliary activities), highlighting a counter-intuitive, positive role of this sugar on the basal expression of these specific genes. As a reminder, we observed the opposite trend for genes encoding pectinases, demonstrating once again the strong dependence of these regulatory mechanisms on the promoter context.

The atypical regulatory patterns observed for several “gene-condition” pairs hence challenged the presence of CreA binding motifs in the promoter region of these genes, (average of 1.7 CreA binding motif per promoter region for the whole selection of GOIs). Especially for cellulases upon glucose starvation, the opposite regulatory behaviour might have been expected according to fungal literature on the cellulolytic system (see above), and because they contain an average of 2.2 consensus per gene promoter, which statistically favours a situation where genes that contain multiple adjacent motifs in their promoter regions are more likely direct targets of CRE-1 [96]. Also, while genes that belong to clusters 4 and 5 contain, respectively less than 1 and slightly more than two motifs per gene, no clear difference could be noticed in their overall response to this carbon starvation condition. Our results then confirmed that it is difficult to anticipate regulatory patterns just referring to TFs binding-sites identification that can return lots of hits, and, even after experimentation, to correlate in silico data to condition-specific changes in regulatory patterns. Sun et al. also stated that it was not possible to predict whether a promoter would be bound by a TF solely on the presence of such motifs [96]. Sound conclusions on the real involvement of TFs hence require (global) experimental validations, as in *N.* *crassa* where ChIP-PCR of putative target genes showed that CRE-1 binds to only some adjacent 5′-SYGGRG-3′ motifs, suggesting that additional regulatory factors affect the specificity of CRE-1 binding to the promoter [96].

With such a variety of data and regulatory patterns, even for those obtained from closely related fungi as *P. funiculosum*, it is difficult to reconcile some of these observations. An example is the positive effect of glucose on cellulase basal expression (our study), and conversely, a basal expression that is negatively regulated by PfMig1 in the presence of glucose [98]. Mechanistically, such apparent discrepancies may rely on genomic sequence differences between the strains, either acting indirectly on TFs localisation and stability, affinity to binding sites, or on local modifications of chromatin structure [53]. Hence, it might be interesting to decipher the exact interplay between glucose, CreA/Cre1/PfMig1 and possibly other TFs and chromatin modifiers. Our attempts at deleting CreA in *T. versatilis* were unfortunately unsuccessful, preventing further investigations on growth phenotypes and the analysis of regulatory patterns in this mutant strain. We faced a similar failure trying to delete the sole zinc finger domain of this transcription factor, while this mutation was successfully obtained in the *P. funiculosum* NCIM1228 strain by using a similar HR-based genetic manipulation [98]. Such technical bottleneck should be alleviated by the implementation of the CRISPR/cas9 system in *T. verstilis*, as it recently proved its efficiency in genome editing in many filamentous fungi species [106–108]. Either for new CreA deletion attempts, or for easier relief from CCR by promoter engineering, of either direct targets of interest or key TFs that might be themselves under the control of CCR [100, 101, 109].

### Gene redundancy in functional categories: the heterogeneity of expression patterns seems to be the rule

A striking feature in the genome of filamentous fungi, including *T.* *versatilis*, is the remarkable redundancy in genes coding for the same enzymatic activity [25–28]. In our non-exhaustive set of GOIs, 8 encode xylanases, 7 of them being members of the GH11 CAZY family. Even more noticeable, 10 encode arabinofuranosidases distributed in CAZY families GH43 (1), GH51 (1), GH54 (5) and GH62 (3), and 11 encode cellulases quite evenly distributed in as much as 7 CAZY families. This redundancy likely goes with slightly different catalytic properties of these enzymes, as for GH54 and GH62 type arabinofuranosidases [110], GH10 and GH11 xylanases [111, 112], or for the cellulolytic activities of multi-enzymatic complex system [113] from *Penicillium funiculosum*.

Data collected in this study clearly confirmed that classification of proteins according to enzymatic activity does not correlate, for the essential, to uniform expression patterns of the different members. Such a variability has been noticed for a long time, studying specific targets as *T.* *reesei xyn1* and *xyn2* as a function of several inducing substrates [114]. In our study, we found an incredible diversity of responses within categories, from variable fold-change values to opposite regulatory patterns between the genes, with strong dependence upon the nutritional environment. Also and despite similar FC values under a specific condition, 3- to 4-log differences in transcript levels were observed, leading possibly to great differences in the levels of expressed proteins, and ultimately in the corresponding hydrolytic activities for a given substrate. Finally, another source of variability came from the contribution of transcription factors, as XlnR, whose impact on gene expression was both gene-specific and culture condition-dependent.

In a similar work, Mikus et al. [115] specifically focused on the expression patterns of different class II hydrophobin genes from *Hypocrea atroviridis*, under various physiological conditions. They also found that the 10 hydrophobins display different patterns of response to these conditions, illustrating both divergent and redundant functions of these family members. Also, comparative studies between eight mycorrhizal species, with 3 up to 27 hydrophobin genes *per* genome, confirmed that the fine-tuning of the expression of these genes in various conditions differed considerably between genes in a species of interest, and further showed differentiation between species without following patterns that would be explained by species traits or ecology [116]. Taken altogether, if considering the whole set of gene-condition pair possibilities available in this study, even within the most homogeneous categories (e.g. cellulases) and particularly for the least homogeneous ones (e.g. arabinofuranosidases), the diversity of response seems to be the rule, while common and stable behaviour between members, the exception.

## Conclusions

Amongst the numerous microorganisms exhibiting the capability of biomass degradation, the filamentous fungi *A. niger* and *T. reesei* (*H. jecorina*) stand as pillars of hydrolytic enzymes production. In the present study, we confirmed the extraordinary potential of *Talaromyces versatilis* (formerly known as *Penicillium funiculosum*), another efficient enzyme producer used at the industrial scale for feed. With few exceptions of conserved regulatory patterns as compared to other enzyme producers, our results emphasized instead the outstanding complexity and divergence of responses, between gene family members, and beyond, with the corresponding categories of genes in more or less distant fungi. This plasticity of gene expression clearly relied on the strong influence of both the inducer and the promoter context, for which lots of factors should ideally be considered in addition to dedicated TFs binding sites, as epigenetic effects, sequence nucleotide environment or presence of associated motifs (review from [117]). Further, on the evidence that TFs rarely trigger transcriptional responses as lonesome and direct effectors, but rather act in concert with multiple factors [34], all these elements actually preclude any anticipation of the transcriptional response of a gene, or subsets of genes, in a specific nutritional context. The rational engineering of a fungus of interest and/or the setup of a new biotechnological process to reach optimized, if not customized expression patterns of enzymes, hence appear almost impossible just relying on published data that can lead, in the best cases, to approximate trends. Preliminary, experimental assays carried out in the context of interest, seem therefore mandatory before thinking in (genetic) strategies for the improvement of enzyme production in fungi.

## Methods

### Strains and culture conditions

The industrial strain *Talaromyces versatilis* IMI 378536, deposited in the International Mycological Institute Strain Collection, is a property of ADISSEO FRANCE SAS (patent W0 99/57325 and US 2003/0108642 A1). It has been isolated after UV mutagenesis from the wild type IMI 134755 strain, and is used for the industrial production of the Rovabio^®^ Excel enzymatic cocktail that is enriched for endoxylanases and cellulases [35]. Strains with deletion of *xlnR* (*ΔxlnR*) or overexpression of this gene (referred as *xlnR*^+^ in this study, *id.* to DSM26702 used for the industrial production of the Rovabio^®^ Advance cocktail) were obtained from the IMI 378536 strain, following the protoplasts transformation method described in [67]. The analysis of *T.* *versatilis xlnR*^+^ genomic DNA by southern blot and digital droplet PCR, (carried out on the Get platform from Génotoul), showed that this mutant strain carries nine copies of *xlnR* under the control of its own promoter, with random integration of a *xlnr* PCR fragment in its genome.

Spores were obtained by growing *T. versatilis* strains on Potato Dextrose Agar (PDA) plates. They were used to inoculate a minimal medium (MM) that contained for 1 L: 1.9 g KH_2_P0_4_, 0.65 g KCl, 0.65 g MgSO_4_, 12.5 mg ZnSO_4_, 12.5 mg MnCl_2_, 12.5 mg FeSO_4_, 5 g NH_4_Cl. Unless otherwise stated, the pH was adjusted to 6.0 with 50 mM KH_2_P0_4_ and MM contained glucose at 10 g/L and was supplemented with 10 mM uridine to allow growth of the *T. versatilis ΔxlnR* since this strain is deleted for the *pyrG* locus. This medium was inoculated with 2 × 10^5^ spores/mL and the cultures was performed at 30 °C in Erlenmeyer flasks on a rotary shaker set at 150 rpm.

To expose *T.* *versatilis* strains to different nutritional conditions, the mycelium obtained after 48 h in MM broth with glucose was filtered through Miracloth (Merck), washed with MM without carbon source and then transferred for the indicated time to fresh media containing the desired carbon source (Table 2). The wild-type and *xlnR*^+^ *T. versatilis* strains were also grown under lab-scale industrial condition that is used for the Rovabio^®^ production, as described in [37]. This fermentation medium is a mix of cellulose and hemicellulose specifically designed for the production of the enzymatic cocktail by *T.* *versatilis* at industrial scale.

### Mycelia samples, RNA extraction and cDNA synthesis

For transcriptomic analysis, samples of about 50 mg of mycelium were collected by filtration through Miracloth and flash frozen in liquid nitrogen. These frozen samples were mechanically disrupted using the TissueLyser II (Qiagen) in the presence of a 5 mm stainless steel bead (Qiagen), with two sequential high-speed shaking, 30 Hz-set cycles of 3 min. Total RNA was isolated from disrupted mycelia samples using the GeneJET Plant RNA Purification Mini Kit (Thermo-Fischer). An on-column DNase I treatment (Thermo) was added to the protocol, applying 100 μL of the DNase I mix (50 μL of DNase I, 10 μL of 10 X buffer and 40 μL of nuclease-free water) to the column after the first wash, for a 30-min incubation at room temperature and final wash with the wash buffer I. The remaining of the protocol, including a second wash and the elution, was performed as recommended by the Supplier.

RNA was quantified using the ND-1000 UV–visible light spectrophotometer (NanoDrop Technologies) and its quality was assessed on a Bioanalyzer 2100 using the RNA 6000 Nano Labchip kit (Agilent). Only RNA samples with 260/280 nm wavelength ratio of approximately 2 and 260/230 nm wavelength ratio greater than 2 were retained for analysis. One microgram of total RNA from mycelia samples were used for the cDNA synthesis reaction using the PrimeScript First Strand cDNA Synthesis Kit (Takara), according to the Manufacturers’ protocol. The cDNA was diluted 1:10 with water and stored at − 20 °C.

### Gene selection and primers design

The choice of a total of 62 genes of interest (GOIs) that encode representative glycoside hydrolases of different GH families is described in the results section. This list of GOIs, manually curated (genome annotation from A. Llanos Ph.D. thesis, (http://www.theses.fr/2014ISAT0029), proprietary data from ADISSEO France SAS), is reported in Table 2. Besides GOIs, 6 other genes, (*ubc6*, *sac7*, *psm1*, *npl1*, *spo7* and *spt3*), were used as reference genes for RT-qPCR data quantification and normalization [59].

**Table 2:** Growth conditions

Condition	Sample	Harvest time (h)	Medium composition	Substrate (reference)
Glucose	AL1	48	MM + glucose 1%	
Cellobiose	AL3	2	MM + cellobiose 0.2%	d-(+)-Cellobiose (Sigma Aldrich—22150)
Avicel	AL18	24	MM + avicel 1%	Avicel PH101 (Sigma Aldrich—11365)
xylan	AL20	24	MM + xylan 1%	Beechwood xylan (Sigma Aldrich—X4252)
wheat straw^a^	AL22	24	MM + wheat straw 1%	Ball-milled wheat straw
N starvation	AL29	1	MM w/o nitrogen + glucose 1%	
C starvation	AL30	1	MM (w/o carbon source)	
Xylose 0.2%	AL38	2	MM + xylose 0.2%	d-(+)-Xylose (Sigma Aldrich no 95729)
Xylose 1%	AL39	1	MM + xylose 1%	
Arabinose 0.2%	AL41	2	MM + arabinose 0.2%	d-(L)-arabinose (Sigma Aldrich—10850)
Arabinose 1%	AL42	1	MM + arabinose 1%	
Soybean^b^	AL43	24	MM + soybean 1%	Ball-milled soybean press cake
Fermentation^c^	AL49	140	Cellulose and hemicellulose mix	

^a^[67]

^b^[118]

^c^Lab-scale industrial condition, carried out at 30 °C, 500 RPM, Ph 4 (proprietary medium composition, ADISSEO FRANCE SAS)

Primers were designed using Vector NTI advance v11 (Life Technologies) with melting temperature of 58─60 °C, length of 18─25 bp and GC content of 50–60%. Most of the genes possess one to several introns in their sequence, which allowed designing the primers at the exon-exon junctions to minimise the amplification of contaminant gDN*A.* Specificity of the primers for each transcript was checked by BLAST analysis against the *T. versatilis* genome. Amplicon sizes ranged between 70 and 230 bp. Reaction efficiency for each pair of primers was tested by the dilution series method using a mix of cDNA samples as the template. The detailed list of the genes, with corresponding sequences and primers, as well as primer efficiencies and amplicon lengths, is available in the Additional file 1.

### Quantitative PCR on the Biomark

High-throughput quantitative PCR (qPCR) was performed using the Fluidigm^®^ Biomark microfluidic system. Every sample-gene combination was analysed using 96.96 Dynamic Array™ Integrated Fluidic Circuits (IFCs), which ensure performing up to 9216 parallel qPCR reactions in nL-scale volumes. Prior to qPCR, the cDNA samples were pre-amplified (activation at 95 °C for 10 min and 14 PCR cycles of 95 °C for 15 s and 60 °C for 4 min) with the PreAmp Master Mix (Fluidigm^®^) and a primer mix at 200 nM, which contains all the primers targeting all the genes analyzed on the array. The samples were treated with Exonuclease I (New England BioLabs), for 30 min at 37 °C; 15 min at 80 °C; and 4 °C hold, and then diluted by adding 18 µL of TE-low EDTA buffer (10 mM Tris–HCl, 0.1 mM EDTA, pH 8). Two microliters of these pre-amplified cDNA samples were mixed with 6 µl of reagent mix into the 96-wells of the Sample plate. This reagent mix consisted of 440 μL 2X TaqMan Master Mix (Applied Biosystems - 430976), 44 μL 20 × DNA Binding Dye Sample Loading Reagent (Fluidigm^®^ - 100-3738), 44 µl 20X Evagreen (Biotium - 31000) plus 132 μL TE. The Sample plate was briefly vortexed and centrifuged. Relative to primer sets preparation, each pair of primers was prepared as a 5 μM solution (each primer) in the Assay loading reagent (Fluidigm^®^) and dispensed into the 96-wells of the Assay plate. Following priming of the Dynamic Array™ IFC in the IFC Controller HX, 5 μL of cDNA samples and primer sets from the Sample and Assay plates, respectively, were dispensed into the inlets of the IFC. After loading the Array using the IFC controller, the IFC was placed into the Biomark for qPCR. The following protocol was used: Thermal mix steps for 2 min at 50 °C; 30 min at 70 °C; 10 min at 25 °C; Hot start for 2 min at 50 °C and 10 min at 95 °C; followed by 35 cycles of 15 s at 95 °C and 1 min at 60 °C. A melting curve at the end of the protocol verified the specificity of the PCR, absence of contamination and of primer dimers. Data was collected with Fluidigm^®^ Real-Time PCR analysis software using the linear baseline correction method and global auto Cq threshold method. Cq values that exceeded 35 cycles were considered beyond the limit of detection. Each array included a negative control (NTC), a positive control for the samples (human gDNA) and a control for the primers (RNAseP).

### Fold-change calculation and statistical analysis

Quantification of the transcripts by RT-qPCR was performed from three independent biological replicates (samples) for each of the 13 culture conditions. A technical duplicate was carried out for each sample, except for the glucose samples that were analyzed in technical quadruplicates due to the low transcript levels of most of the GOIs under this condition. Raw qPCR signals were analyzed using the Real-Time PCR Analysis software (Fluidigm^®^) to determine Cq values. These Cq were then used to calculate the Fold-Change (FC) values, taking into consideration each primer pair efficiency. Data normalization was performed using a multiple reference genes procedure, with the geometrical mean of 6 validated reference genes for *T. versatilis*, i.e. *ubc6*, *sac7*, *psm1*, *npl1*, *spo7* and *spt3* [59]. FC statistics (mean ± SD) were determined from the 3 biological replicates. Unless otherwise stated, the values were expressed as log_2_ (FC). Statistical analyses and advanced graphics were conducted using R (https://www.r-project.org/). The full set of expression data used in this article, for the three strain backgrounds, is provided in the Additional file 8.

### Upstream regulatory motifs analysis

Upstream regions of the genes of interest, (700 bp upstream of the ATG codon), were analysed for the presence of *cis*-acting elements using RSAT Fungi (Regulatory Sequence Analysis Tool—http://rsat-tagc.univ-mrs.fr/rsat/). Each complementary strand of the promoter was scanned for the motifs 5′-GGCTAA-3′ (XlnR), 5′-GGCWAW-3′ (alternative XlnR motif) and 5′-SCGGRG-3′ (CreA) (Additional file 1). The analysis was also performed with the MEME suite (Multiple EM for Motif Elicitation—http://meme-suite.org) to confirm the results.

## Additional files

**Additional file 1.** Detailed information on GOIs. Gene annotations, qPCR primers specifications and CDS sequence, number of each of TF binding motifs in the promoter region, FC values for the ‘glucose repression’ condition (extracted from our previous RNA-seq study [59]).

**Additional file 2.** Expression profiles in gene clusters, in the WT strain, as a function of culture conditions. Radar chart representing the expression level of GOIs (log_2_ (FC) values), in each of the five gene clusters defined in Fig. 2. Each axis corresponds to a culture condition, as defined in the bottom right legend of the figure. The coloured line represents the average log_2_ (FC) value of GOIs gathered in the cluster.

**Additional file 3.** GOIs expression data in each gene cluster. Box plot illustration of log_2_ (FC) values as a function of the culture conditions and in the three strain backgrounds (grey for the WT, green for the *ΔxlnR* mutant, and red for the *xlnR*^+^ strain). The ordering of conditions (from the left (Straw) to the right (N-starv)) relies on Fig. 2 classification (top-down order).

**Additional file 4.** GOIs expression data in each functional category. Box plot illustration of log_2_ (FC) values as a function of the culture conditions and in the WT, *ΔxlnR* and *xlnR*^+^ strains. Legend as in Additional file 3.

**Additional file 5.** Specificity of the *ΔxlnR* response patterns on xylose, xylan and straw. Substrate-dependent scatter plots of log_2_ (FC) values in the *ΔxlnR* strain as a function of log_2_ (FC) values in the wild type strain. The GOIs are gathered according to the five gene clusters that have been defined in Fig. 2.

**Additional file 6.** Specificity of the *xlnR*^+^ response patterns on xylose, xylan and straw. Substrate–dependent scatter plots of log_2_ (FC) values in the *xlnR*^+^ strain as a function of log_2_ (FC) values in the wild type strain. The GOIs are gathered according to the five gene clusters that have been defined in Fig. 2.

**Additional file 7.** Pairwise correlations between genes encoding backbone hydrolases, in the three strain backgrounds. These matrices were drawn using expression values from Fig. 2 (**a**; WT strain), Fig. 4 (**b**; *ΔxlnR* strain) and Fig. 5 (**c**; *xlnR*^+^ strain). Same legend as in Fig. 7. We arbitrary coloured in green the pairs that exhibited rather good pairwise correlations in the WT strain (*r *> 0.90).

**Additional file 8.** Data file of expression data from the study. This xlsx file contains all calculated Log2(FC) values from this study, used for graphics preparation, and that can be readily imported in dedicated statistical analysis softwares (e.g. R, Prism, Statgraphics, etc.). Use the *Read Me* sheet for detailed information on the organisation of the data in the different sheets of the file.

## Abbreviations

CAZyme: carbohydrate-active enzymes
CCR: carbon catabolite repression
Cq: quantification cycle in qPCR
C-starv: carbon starvation
ddPCR: digital droplet PCR
FC: fold change
GH: glycosyl hydrolase
GOI: Gene Of Interest
HR-based genetic manipulation: Homologous Recombination-based genetic manipulation
log_2_ (FC): logarithm base 2 of fold change values
MM: minimal medium
N-Starv: nitrogen starvation
qPCR: quantitative PCR
RNA-seq: whole cell RNA sequencing
RPKM: reads per kilobase of exon model per million mapped reads
RQ: relative quantity
TF: transcription factor
