# Supplementary material for: Carbon sources and XlnR-dependent transcriptional landscape of CAZymes in the industrial fungus Talaromyces versatilis: when exception seems to be the rule
Source: Microb Cell Fact. 2019 Jan 28;18:14. doi: 10.1186/s12934-019-1062-8 (PMC6348686; doi:10.1186/s12934-019-1062-8)
Supplement: Supplementary file 2 — Additional file 2. Expression profiles in gene clusters, in the WT strain, as a function of culture conditions. Radar chart representing the expression level of GOIs (log2 (FC) values), in each of the five gene clusters defined in Fig. 2. Each axis corresponds to a culture condition, as defined in the bottom right legend of the figure. The coloured line represents the average log2 (FC) value of GOIs gathered in the cluster. [file 12934_2019_1062_MOESM2_ESM.pdf]

Additional file 2: Figure S1

Cluster 1

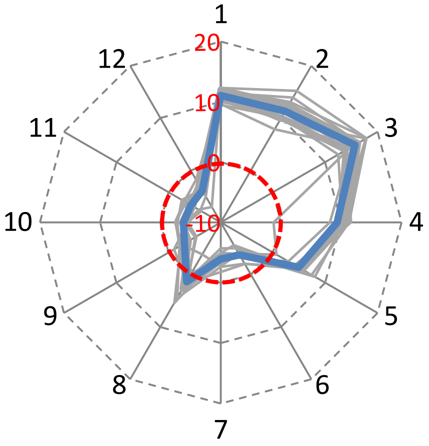

Cluster 2

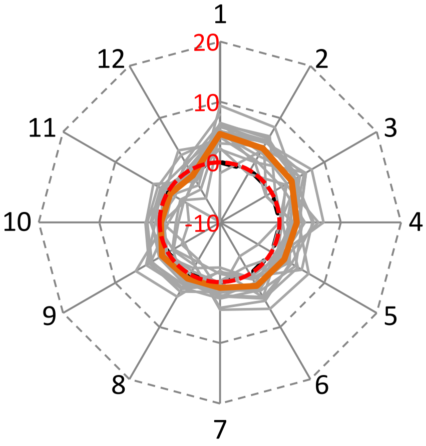

Cluster 3

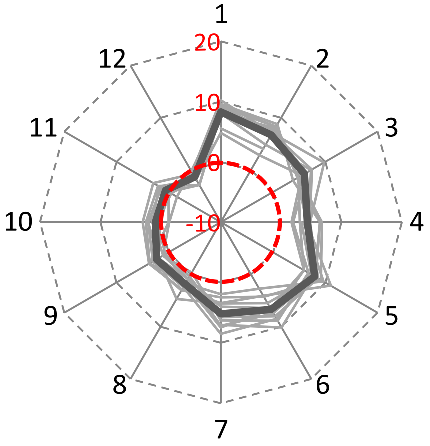

Cluster 4

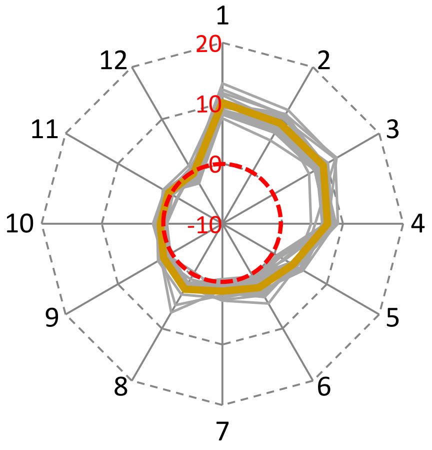

Cluster 5

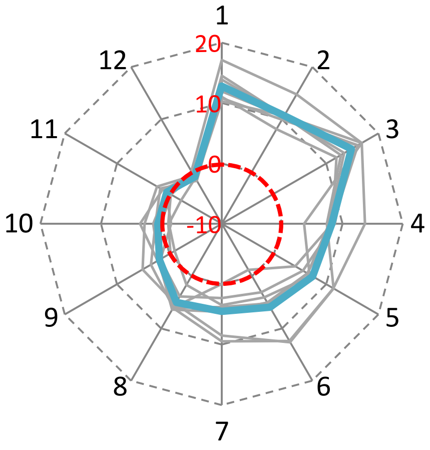

Axes (conditions) :

1) Straw 1%
2) Soybean 1%
3) Fermentation
4) Avicel 1%
5) Xylan 1%
6) Xylose 0.2%
7) Xylose 1%
8) Cellobiose 0.2%
9) Arabinose 0.2%
10) C starvation
11) Arabinose 1%
12) N starvation
