# Supplementary figures and images for: Carbon sources and XlnR-dependent transcriptional landscape of CAZymes in the industrial fungus Talaromyces versatilis: when exception seems to be the rule
Source: Microb Cell Fact. 2019 Jan 28;18:14. doi: 10.1186/s12934-019-1062-8 (PMC6348686; doi:10.1186/s12934-019-1062-8)

# Additional file 3: Figure S2

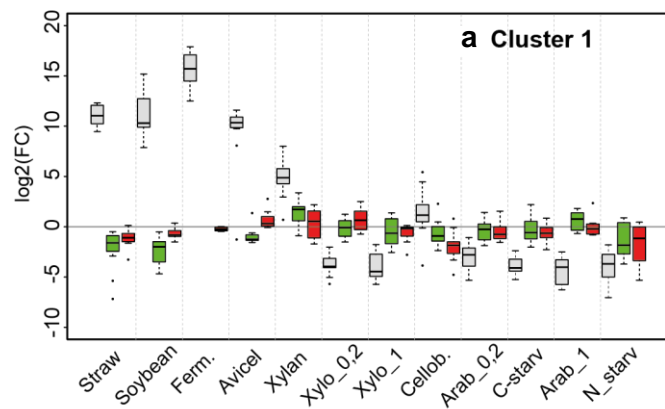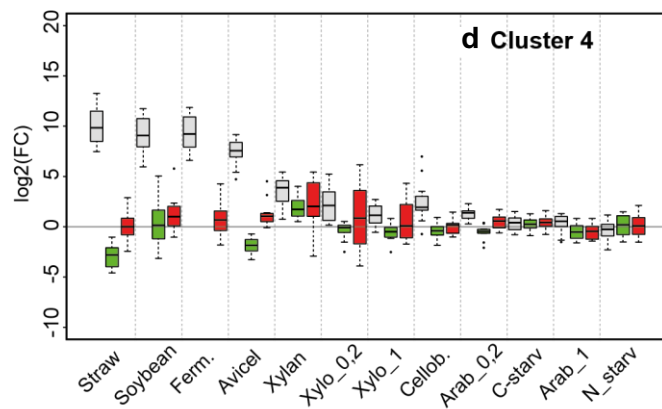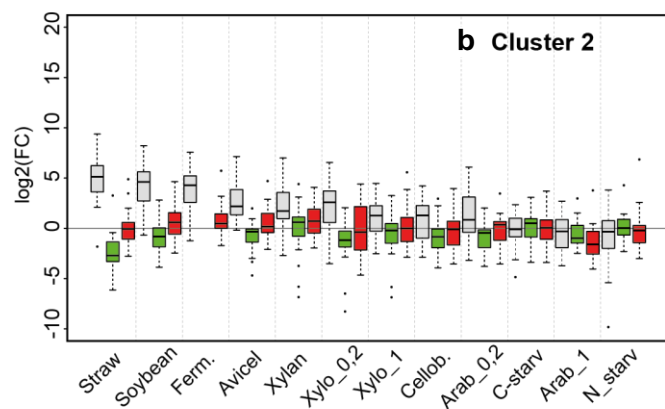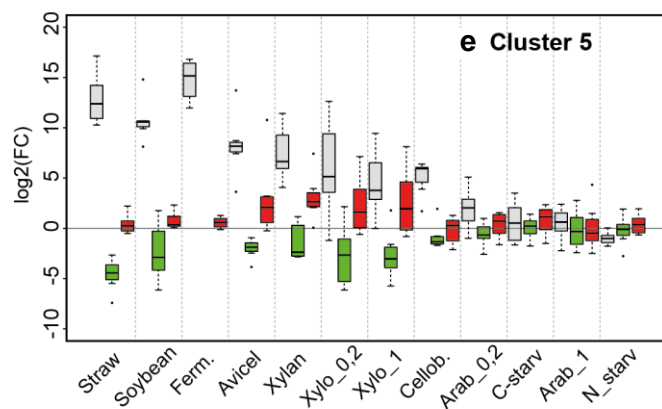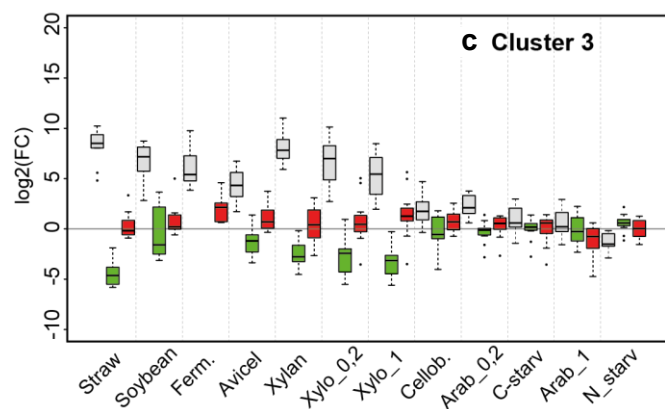

Supplement: Supplementary file 3 — Additional file 3. GOIs expression data in each gene cluster. Box plot illustration of log2 (FC) values as a function of the culture conditions and in the three strain backgrounds (grey for the WT, green for the ΔxlnR mutant, and red for the xlnR+ strain). The ordering of conditions (from the left (Straw) to the right (N-starv)) relies on Fig. 2 classification (top-down order). [file 12934_2019_1062_MOESM3_ESM.pdf]

# Additional file 4: Figure S3

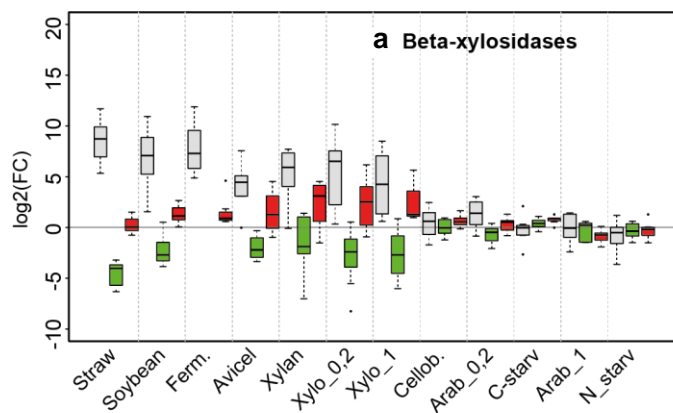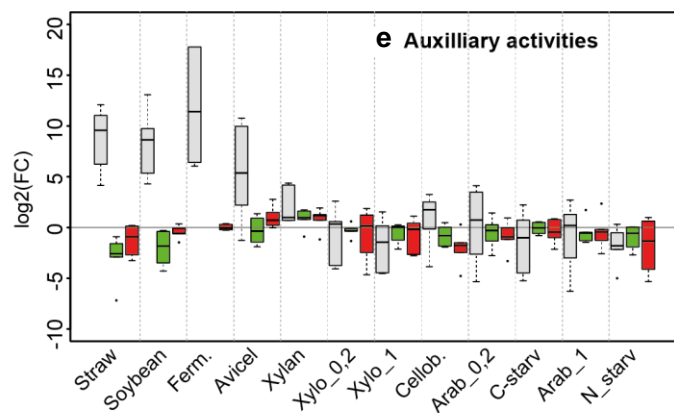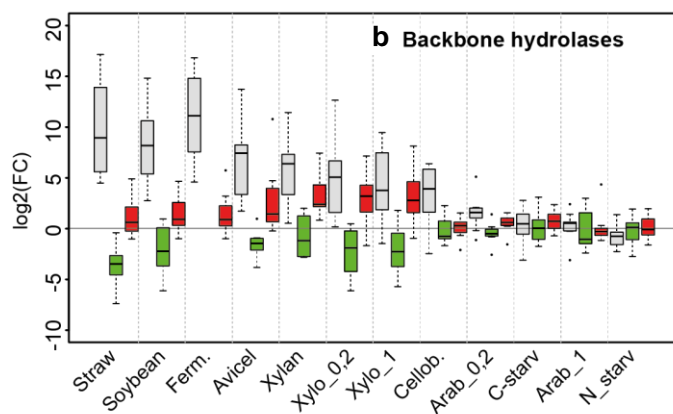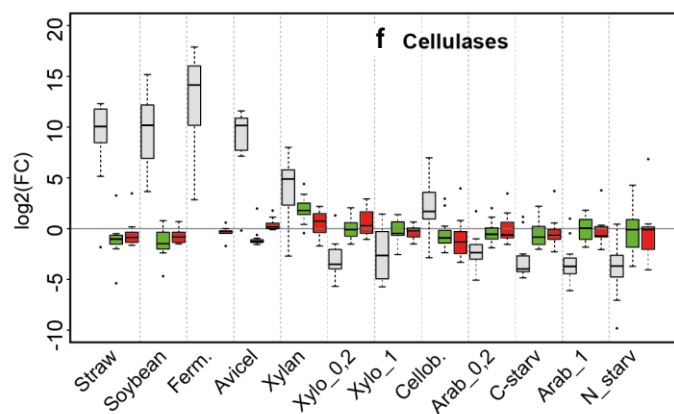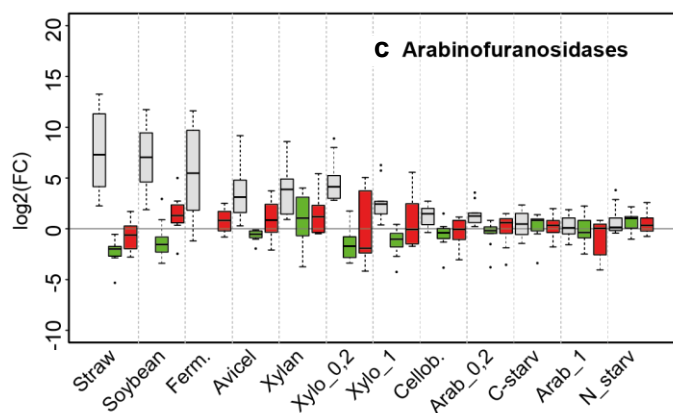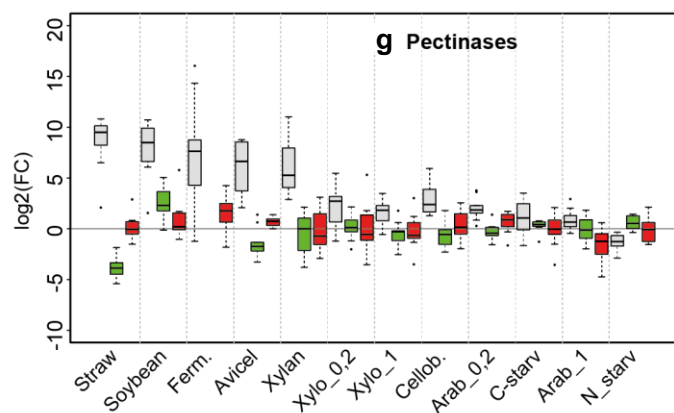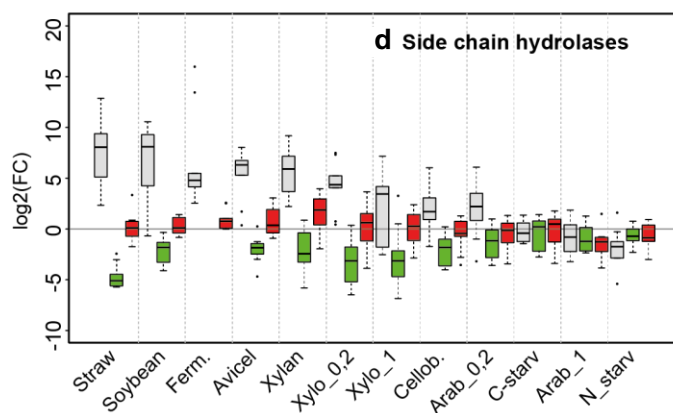

Supplement: Supplementary file 4 — Additional file 4. GOIs expression data in each functional category. Box plot illustration of log2 (FC) values as a function of the culture conditions and in the WT, ΔxlnR and xlnR+ strains. Legend as in Additional file 3. [file 12934_2019_1062_MOESM4_ESM.pdf]

# Additional file 5: Figure S4

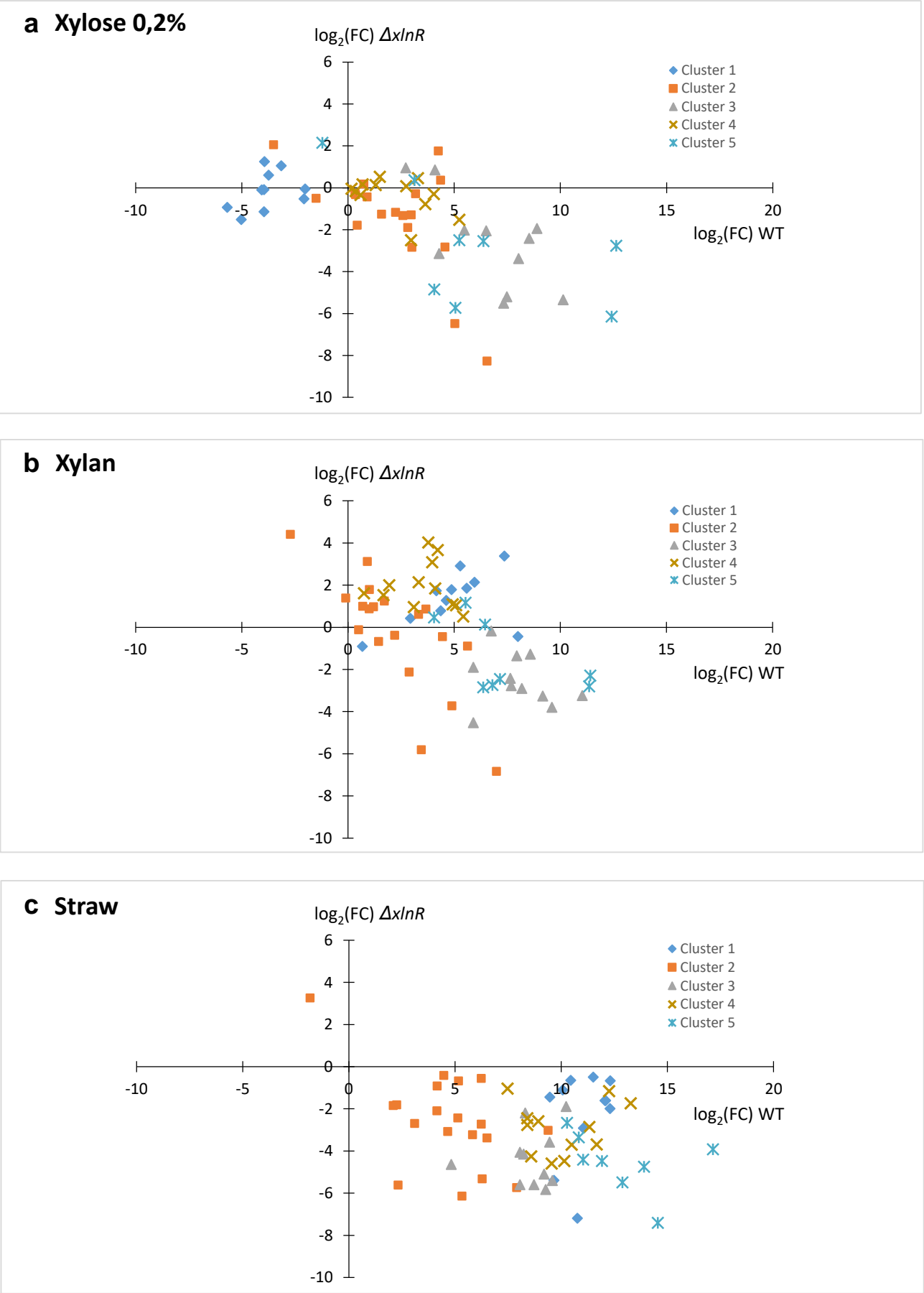

Supplement: Supplementary file 5 — Additional file 5. Specificity of the ΔxlnR response patterns on xylose, xylan and straw. Substrate-dependent scatter plots of log2 (FC) values in the ΔxlnR strain as a function of log2 (FC) values in the wild type strain. The GOIs are gathered according to the five gene clusters that have been defined in Fig. 2. [file 12934_2019_1062_MOESM5_ESM.pdf]

# Additional file 6: Figure S5

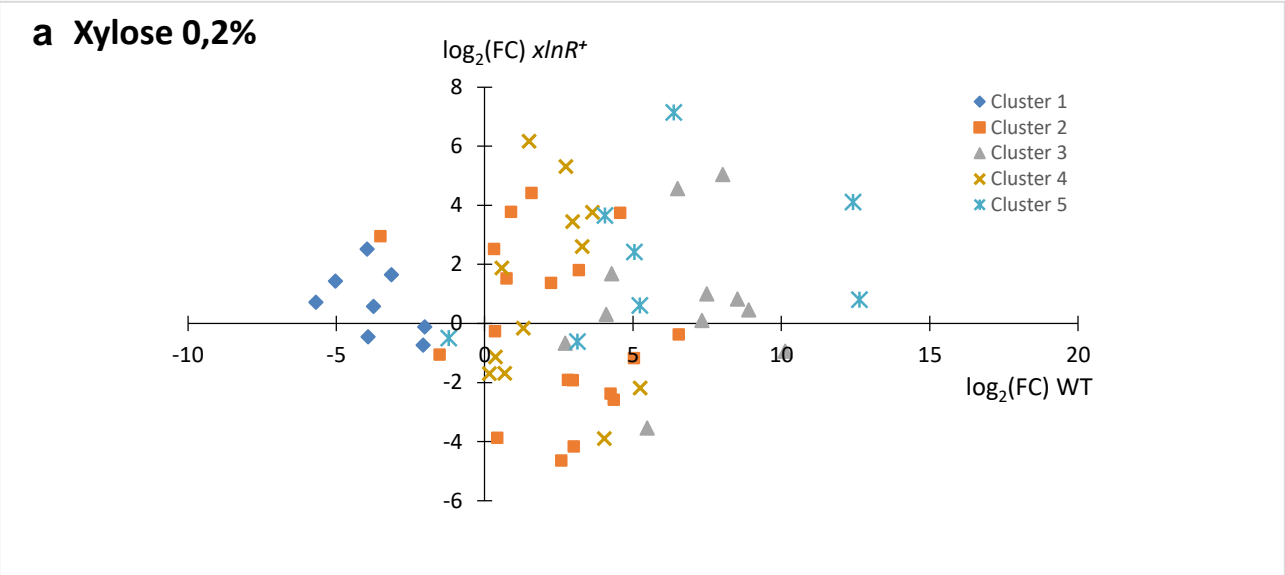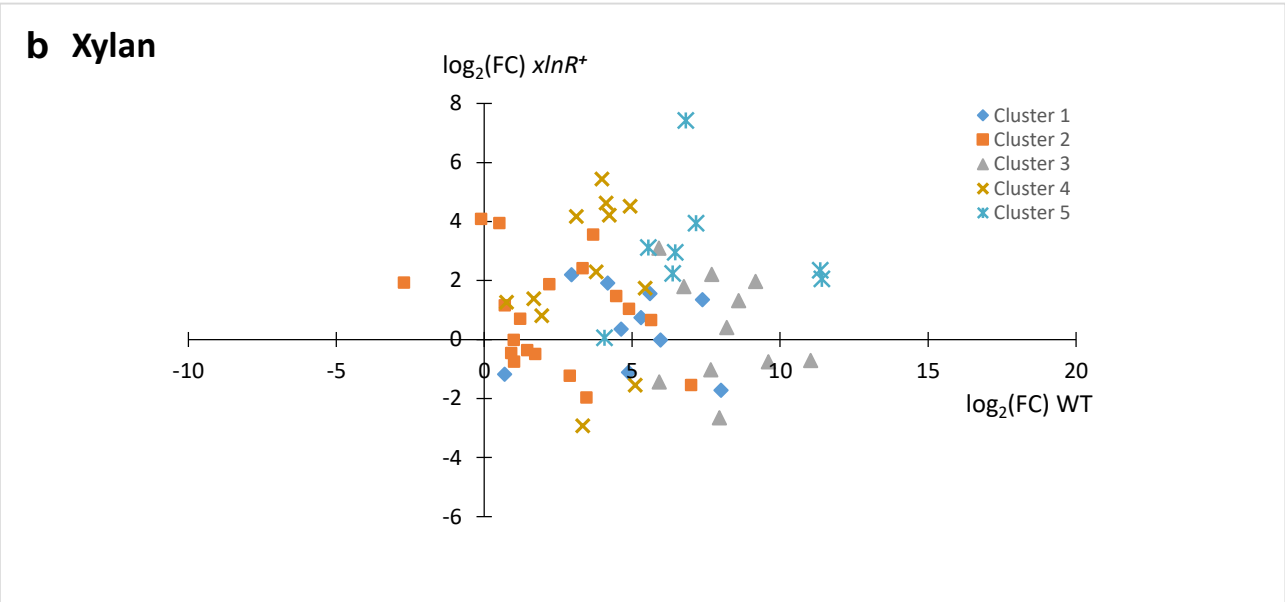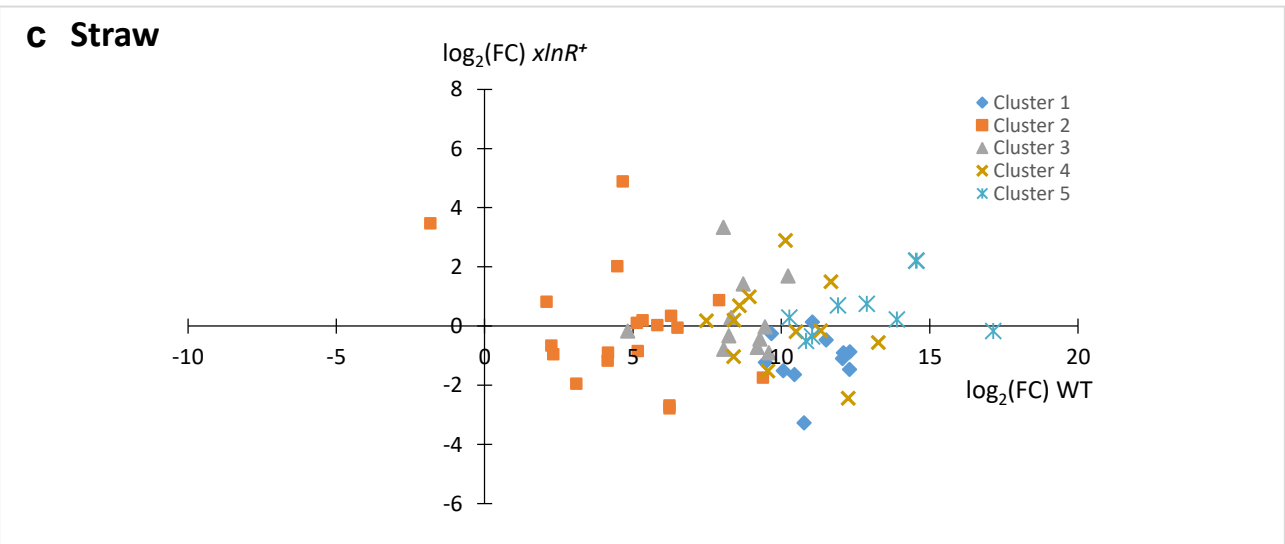

Supplement: Supplementary file 6 — Additional file 6. Specificity of the xlnR+ response patterns on xylose, xylan and straw. Substrate–dependent scatter plots of log2 (FC) values in the xlnR+ strain as a function of log2 (FC) values in the wild type strain. The GOIs are gathered according to the five gene clusters that have been defined in Fig. 2. [file 12934_2019_1062_MOESM6_ESM.pdf]

Additional file 7: a

Figure S6

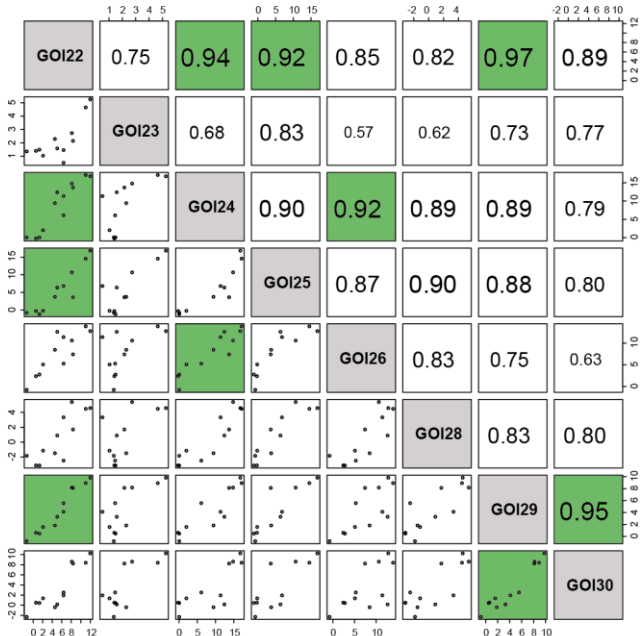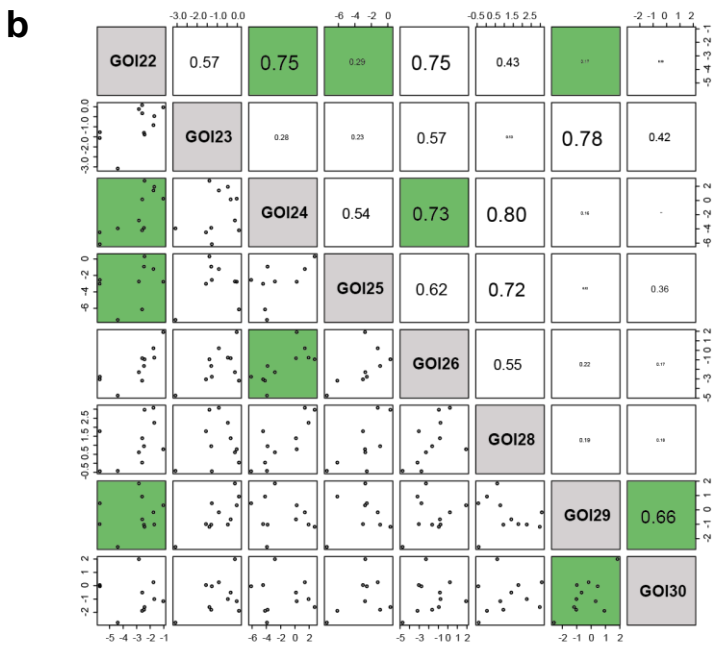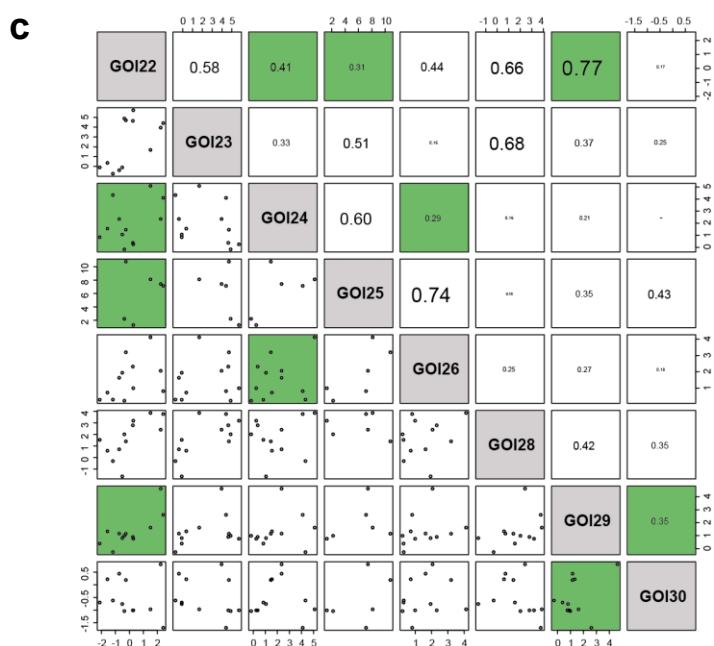

Supplement: Supplementary file 7 — Additional file 7. Pairwise correlations between genes encoding backbone hydrolases, in the three strain backgrounds. These matrices were drawn using expression values from Fig. 2 (a; WT strain), Fig. 4 (b; ΔxlnR strain) and Fig. 5 (c; xlnR+ strain). Same legend as in Fig. 7. We arbitrary coloured in green the pairs that exhibited rather good pairwise correlations in the WT strain (r > 0.90). [file 12934_2019_1062_MOESM7_ESM.pdf]
